# Prostate-specific membrane antigen radioguided surgery with negative histopathology: an in-depth analysis

**DOI:** 10.1007/s00259-023-06442-7

**Published:** 2023-09-26

**Authors:** Daniel Koehler, Samuel Trappe, Farzad Shenas, Amir Karimzadeh, Ivayla Apostolova, Susanne Klutmann, Francesca Ambrosini, Lars Budäus, Fabian Falkenbach, Sophie Knipper, Tobias Maurer

**Affiliations:** 1 Department of Diagnostic and Interventional Radiology and Nuclear Medicine, University Medical Center Hamburg-Eppendorf, 20246 Hamburg, Germany; 2 Martini-Klinik Prostate Cancer Center, University Hospital Hamburg-Eppendorf, Hamburg, Germany; 3 IRCCS Ospedale Policlinico San Martino, Genoa, Italy; 4 Department of Urology, University Hospital Hamburg-Eppendorf, Hamburg, Germany

**Keywords:** Prostate cancer, PSA recurrence, PSMA PET/CT, PSMA-radioguided surgery

## Abstract

**Purpose:**

To identify reasons for negative histopathology of specimens from prostate-specific membrane antigen (PSMA) radioguided surgery (PSMA-RGS) in recurrent prostate cancer (PCa) after prostatectomy.

**Methods:**

Of 302 patients who underwent PSMA-RGS, 17 (5.6%) demonstrated a negative histopathology. Preoperative data, PSMA PET, PSMA SPECT, and follow-up information were analyzed retrospectively to differentiate true/false positive (TP/FP) from true/false negative (TN/FN) lesions.

**Results:**

The median prostate-specific antigen at PET was 0.4 ng/ml (interquartile range [IQR] 0.3–1.2). Twenty-five index lesions (median short axis 7 mm, IQR 5–8; median long-axis 12 mm, IQR 8–17) had a median SUVmax of 4 (IQR 2.6–6; median PSMA expression score 1, IQR 1–1). Six lesions were TP, twelve were FP, one was TN, and six remained unclear. All TP lesions were in the prostatic fossa or adjacent to the internal iliac arteries. Three suspected local recurrences were FP. All FP lymph nodes were located at the distal external iliac arteries or outside the pelvis. A low PSMA-expressing TN node was identified next to a common iliac artery. Unclear lesions were located next to the external iliac arteries or outside the pelvis.

**Conclusion:**

In most cases with a negative histopathology from PSMA-RGS, lesions were FP on PSMA PET. Unspecific uptake should be considered in low PSMA-expressing lymph nodes at the distal external iliac arteries or outside the pelvis, especially if no PSMA-positive lymph nodes closer to the prostatic fossa are evident. Rarely, true positive metastases were missed by surgery or histopathology.

**Supplementary Information:**

The online version contains supplementary material available at 10.1007/s00259-023-06442-7.

## Introduction

Radical prostatectomy or radiotherapy (i.e., external beam radiation or brachy therapy) represents the standard of care therapy options with curative intent in prostate cancer (PCa) [1]. Disease recurrence, usually detected by rising prostate-specific antigen (PSA) values, occurs frequently after primary therapy [2–6]. It can be an independent risk factor for PCa-specific mortality, especially in patients with a high Gleason grade, a short interval from primary treatment to biochemical failure, and a short PSA doubling time [7]. Prostate-specific membrane antigen (PSMA) targeting PET/CT has demonstrated high sensitivity and specificity with a substantial impact on clinical decision-making in patients with PSA relapse [8–11]. PSMA PET offers detection rates between 30 and 50%, even at low PSA values of <0.5 ng/ml [12, 13]. The current guideline of the European Association of Urology recommends its use in patients with biochemically recurrent PCa if the results will influence subsequent treatment decisions [1]. The improved detection of recurrent disease has fueled further developments in the field of metastasis-directed therapy strategies like salvage radiation or salvage lymph node dissection [14]. Recent studies showed that PSMA imaging-guided radiotherapy of patients with oligorecurrence on PSMA PET/CT could delay disease progression [15–17]. Alternatively, PSMA-radioguided surgery (PSMA-RGS) represents a metastasis-directed therapeutic strategy which utilizes γ-emitting radioligands to identify PSMA-positive lesions intraoperatively [18–21]. While conventional salvage lymph node dissection showed negative pathological results of the resected specimens in roughly 20% of cases [22, 23], PSMA-RGS was able to improve intraoperative lesion identification, reducing that rate to almost 5% [24]. However, negative surgical results remain in a subgroup of patients.

The aim of this study was to identify reasons for negative histopathological results of specimens from PSMA-RGS in patients with suspected oligorecurrent PCa.

## Materials and methods

### Study population

Case data of 302 patients who underwent PSMA-RGS between January 2018 and December 2021 at a tertiary center were analyzed retrospectively. The presented cohort is a subgroup of a previous publication [24]. Patients were eligible for PSMA-RGS if the following criteria were met: persistent or rising PSA after radical prostatectomy with/without adjuvant or salvage radiotherapy, surgically accessible suspected PSMA-positive local recurrences or PSMA-positive lymph nodes with PSMA expression above the background on PSMA PET, and estimated life expectancy of more than ten years. Patients were not eligible for PSMA-RGS if they had received androgen deprivation therapy in the last six months prior to surgery or demonstrated metastases to supradiaphragmatic lymph nodes, bones, or viscera.

Patients were only considered for the current analysis if the histopathological workup of the resected tissue did not show evidence of PCa, including immunohistochemical staining. Preoperative PSMA PET/CT or PET/MRI and PSMA SPECT/CT had to be available for reassessment. Postoperative clinical data were extracted from the clinical information system (Soarian Clinicals, Cerner Corp., Kansas City, MO, USA) with a follow-up of at least 12 months after PSMA-RGS.

This retrospective analysis was performed in line with the principles of the Declaration of Helsinki. The local institutional review board approved the retrospective single-center investigation and waived the requirement for informed consent (PV7316).

### PSMA PET imaging and analysis

PSMA PET/CT or PET/MRI was performed at different institutions with ^68^Ga- or ^18^F-labeled radioligands (Table 1, Supplementary Table 1). Reports of each scan were reviewed, and PET images were further analyzed by a board-certified radiologist with 4 years of experience in PSMA PET imaging (D.K.). The following parameters were recorded for all lesions of the initial reports (index lesions) and for additional foci that were not described in these reports (additional lesions). Short- and long-axis diameters were measured for each lesion on the axial plane. Radioligand uptake was evaluated qualitatively based on the Prostate Cancer Molecular Imaging Standardized Evaluation (PROMISE V2) criteria (PSMA expression score: 0 = uptake below/equal to blood pool, 1 = uptake above blood pool and lower/equal to liver/spleen, 2 = uptake above liver/spleen and lower/equal to parotid gland, 3 = above parotid gland) [25]. The SUVmax and SUVmean were calculated with isocontours set at 40% of the maximum. Furthermore, the SUVmean of the background (BG) was determined using a 10-ml spherical region of interest drawn in the gluteus muscle. Ratios of the SUVmax and the SUVmean of the BG were calculated for each lesion (SUVmax to BG).

**Table 1:** Imaging characteristics of preoperative PSMA PET scans and [^99m^Tc]Tc-PSMA-I&S SPECT/CT

Patient	Indication PSMA PET/CT	PSA at PET/CT (ng/ml)	Radioligand	Activity PET (MBq)	No. of index lesions	No. of add. lesions revision	Activity SPECT (MBq)	No. of lesions SPECT
1	BCR	1.2	[^18^F]rhPSMA-7	313	2	0	690	0
2	BCR	0.7	[^68^Ga]Ga-PSMA-I&T	159	2	1	759	0
3	BCR	2	[^68^Ga]Ga-PSMA-11	128	1	0	752	1
4	BCR	0.1	[^18^F]PSMA-1007	396	2	0	713	0
5	BCR	1.3	[^18^F]PSMA-1007	267	2	1	630	0
6	BCR	0.4	[^18^F]rhPSMA-7	388	2	0	658	2
7	BCR	11	[^18^F]PSMA-1007	332	1	0	775	0
8	BCR	0.4	[^18^F]PSMA-1007	419	1	3	745	0
9	BCR	1.3	[^68^Ga]Ga-PSMA-I&T	143	1	1	717	0
10	BCR	0.3	[^18^F]PSMA-1007	211	3	0	761	0
11	BCR	0.2	[^18^F]PSMA-1007	312	1	0	826	0
12	BCR	0.2	[^68^Ga]Ga-PSMA-11	71	1	0	766	1
13	BCR	0.5	[^68^Ga]Ga-PSMA-I&T	138	1	0	813	0
14	BCR	0.3	[^18^F]PSMA-1007	478	1	0	750	0
15	BCR	0.7	[^68^Ga]Ga-PSMA-I&T	247	1	1	708	0
16	BCP	0.3	[^68^Ga]Ga-PSMA-I&T	201	1	0	699	0
17	BCR	0.4	[^68^Ga]Ga-PSMA-11	174	2	0	783	1

*BCP*, biochemical persistence; *BCR*, biochemical recurrence; *PSA*, prostate-specific antigen; *PSMA*, prostate-specific membrane antigen

A lesion was interpreted as true positive if it was unchanged or progressive on follow-up PSMA PET (i.e., not removed during PSMA-RGS). Furthermore, if no follow-up imaging was available, a true positive lesion was considered if the postoperative PSA decreased >50%, assuming that the PSMA-positive lesion was not identified in the histopathological workup. Lastly, a lesion was seen as true positive if it demonstrated a radioligand uptake above the background and it was not part of the resection template. False-positive lesions were assumed if foci demonstrated a low PSMA expression (visual score 1), and the postoperative PSA did not decrease after PSMA-RGS, although the lesion was part of the compatible resection template. Furthermore, an alternative PSMA-positive lesion or clinical follow-up data had to indicate another cause of the elevated PSA (e.g., follow-up imaging with another positive lesion, successful salvage therapy of another location). Alternatively, a false-positive lesion was considered if the lesions were incorrectly interpreted as positive due to artifacts (e.g., urine artifact). Lesions were true negative if they were not evident on follow-up imaging, and the PSA remained unchanged or increased after salvage lymphadenectomy with an adequate resection template. No false-negative lesions were encountered because none of the resected specimens yielded malignant cells. Lesions were seen as unclear if patients received no further diagnostic workup after RGS, no other PSMA-positive lesions were found on the revision of the preoperative PSMA PET scans, and no follow-up therapy/imaging indicated the true location of the PSA recurrence.

### PSMA SPECT/CT imaging and analysis

Patients received a median dose of 750 MBq (interquartile range [IQR] 704–771) [^99m^Tc]Tc-PSMA-I&S at a median time of 17.7 h (IQR 17.2–18.2) prior to SPECT/CT. [^99m^Tc]Tc-PSMA-I&S was produced under the conditions of §13 (2b) of the Arzneimittelgesetz (German Medicinal Products Act) as reported previously [19]. SPECT/CT acquisition was performed with a Mediso AnyScan^®^ Trio (Mediso Medical Imaging Systems, Budapest, Hungary) or a Siemens Symbia Intevo™ 16 (Siemens Healthineers, Erlangen, Germany). Whole body planar scans were acquired with 120 mm/min scan speed. SPECT of the abdomen and pelvis were obtained in a 128 × 128 matrix with 96 frames per rotation in 40 seconds per stop (Mediso AnyScan^®^ Trio) or 64 frames per rotation in 20 seconds per stop (Symbia Intevo™ 16). The following CT was conducted with 100–130 kV and an adaptive dose modulation. CT images were obtained in a 512 × 512 matrix and 0.6-mm slice thickness. Data were reconstructed in 3 dimensions with a slice thickness of 3 mm. All lesions that were identified on prior PSMA PET/CT scans were evaluated qualitatively using a visual 4-point scale (0 = no visible uptake, 1 = minimal uptake [only perceivable in knowledge of the PET result], 2 = moderate uptake, 3 = intense uptake).

### PSMA-radioguided surgery and clinical follow-up

All patients were informed regarding the experimental nature of salvage surgery and provided their written informed consent to PSMA-RGS. PSMA-RGS was conducted after a median uptake time of 22 h (IQR 21.1–24.6) of the [^99m^Tc]Tc-PSMA-I&S. The surgical procedure consisted of a template-based lymphadenectomy of the respective side of the pelvis in which the PSMA-positive lesion had been identified on the previous PSMA PET. By the surgeon’s discretion, an extended lymphadenectomy of the contralateral side was possible. If retroperitoneal lesions were evident on imaging, the lymph node dissection template for patients with testicular cancer was employed in addition to the pelvic template. A gamma probe (Crystal Probe CXS-SG603; sensitivity maximum: 13500 cps/MBq, resolution: 14 mm, energy range: 50–511 keV; Crystal Photonics, Berlin, Germany) with acoustic feedback was used for radioactivity measurements. Postoperative complications were assessed according to the Clavien-Dindo classification system [26]. PSA values were measured before PSMA-RGS and at a median of 44 days (IQR 32–143) after PSMA-RGS to determine the biochemical response.

### Statistical analysis

Descriptive statistics were used to illustrate frequencies and proportions for categorical variables. Continuous variables are described with median and IQR. Due to the small patient cohort, no statistical hypothesis testing was conducted. Graphs were created using GraphPad Prism version 9.5.1 (GraphPad Software, San Diego, USA) or SankeyMATIC [27].

## Results

Of 302 patients who underwent PSMA-RGS, histopathology of the resected tissue was negative in 17 cases (5.6%), which were included in the further analysis. In this subgroup, all patients had undergone radical prostatectomy as primary treatment (Table 2) and six patients (patients: 1, 2, 4, 6, 8, 9) also had received adjuvant radiotherapy before the preoperative PSMA PET scans (median time from RP to radiotherapy 11 months, IQR 3–29). PSMA PET was conducted due to biochemical relapse in 16 patients (median time from RP to PSMA PET 59 months, IQR 26–81) and because of biochemical persistence in one patient (patient 16, 3 months after RP).

**Table 2:** Patient characteristics primary therapy

Variable		
PSA at RP (ng/ml)		Median 8.3, IQR 4.6–13.4
RP technique	Open	12 (71%)
	Robotic	5 (29%)
pT-stage RP	T2	8 (47%)
	T3	9 (53%)
Gleason grade group	I	2 (12%)
	II	6 (35%)
	III	8 (47%)
	IV	1 (6%)
Resection margin	R0	11 (65%)
	R1	6 (35%)
pN-stage RP	N0	13 (76%)
	N1	2 (12%)
	Nx	2 (12%)
Adjuvant radiotherapy	Yes	6 (35%)
	No	11 (65%)

*IQR*, interquartile range; *PSA*, prostate-specific antigen; *RP*, radical prostatectomy

### PET and SPECT imaging

The median PSA at PET was 0.4 ng/ml (IQR 0.3–1.2). A total of 25 index lesions were described in the primary reports (median lesions per case 1, IQR 1–2). The median short axis of the encountered foci was 7 mm (IQR 5–8) with a median long axis of 12 mm (IQR 8–17). In cases 4, 9, and 17, the dimensions of the suspected local recurrences were not measurable due to their close vicinity to the surrounding structures. Neither dimensions nor SUV of the suspected lesion in patient 13 was measured because it was seen as a urine artifact on revision (Supplementary Fig. 1). The median PSMA expression score of the index lesions was 1 (IQR 1–1). The median SUVmax was 4 (IQR 2.6–6.0) with a median SUVmax to BG ratio of 9.4 (IQR 6.6–13.2).

Seven additional lesions (22%) that were not described in the primary reports were found in five patients (29%) on review of the preoperative PSMA PET/CT (median SUVmax 5, IQR 4.4–7.1; median visual score 1, IQR 1–2). These lesions included five local recurrences, one locoregional lymph node, and one bone lesion (Supplementary Table 1).

Of 32 lesions on preoperative PSMA PET (including lesions that were only identified on revision), five (16%) were visible on PSMA-I&S SPECT/CT (Supplementary Table 1).

### PSMA-radioguided surgery

In total, 344 lymph nodes (median 19, IQR 12–27) were resected. Patient 16 only received resection of a lesion which was suspicious for a local recurrence/residual tumor of the left seminal vesicle; no lymph nodes were removed. The resection templates of all other patients included pelvic lymph nodes. These were extended to the retroperitoneum in 13 patients (76%, Supplementary Table 1). Surgical complications arose in eleven cases (65%), of which seven (64%) were minor postoperative complications (Clavien-Dindo I–II). Four patients suffered from Clavien-Dindo complications III, including one case of a lymphocele which was drained under CT-fluoroscopic guidance, a patient with a postoperative hematoma, and two patients who suffered from an injury to the ureter. No life-threatening (Clavien-Dindo IV) or fatal complications (Clavien-Dindo V) occurred.

### Clinical follow-up and lesion analysis

**Fig. 1:**
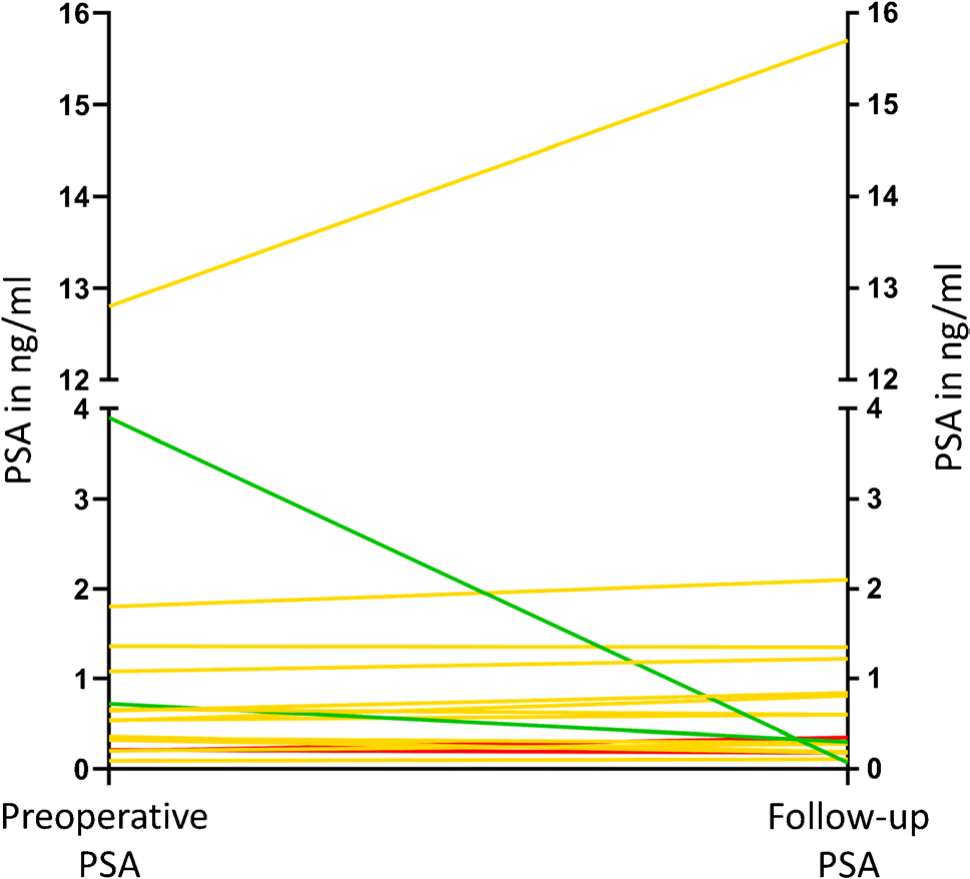
Prostate-specific antigen trend after prostate-specific membrane antigen (PSMA) radioguided surgery (RGS). Spaghetti plot illustrating the preoperative prostate-specific antigen (PSA) value and the follow-up PSA after PSMA-RGS. Green – PSA decrease >50%; yellow – PSA change ≤50%; red – PSA increase >50%

**Fig. 2:**
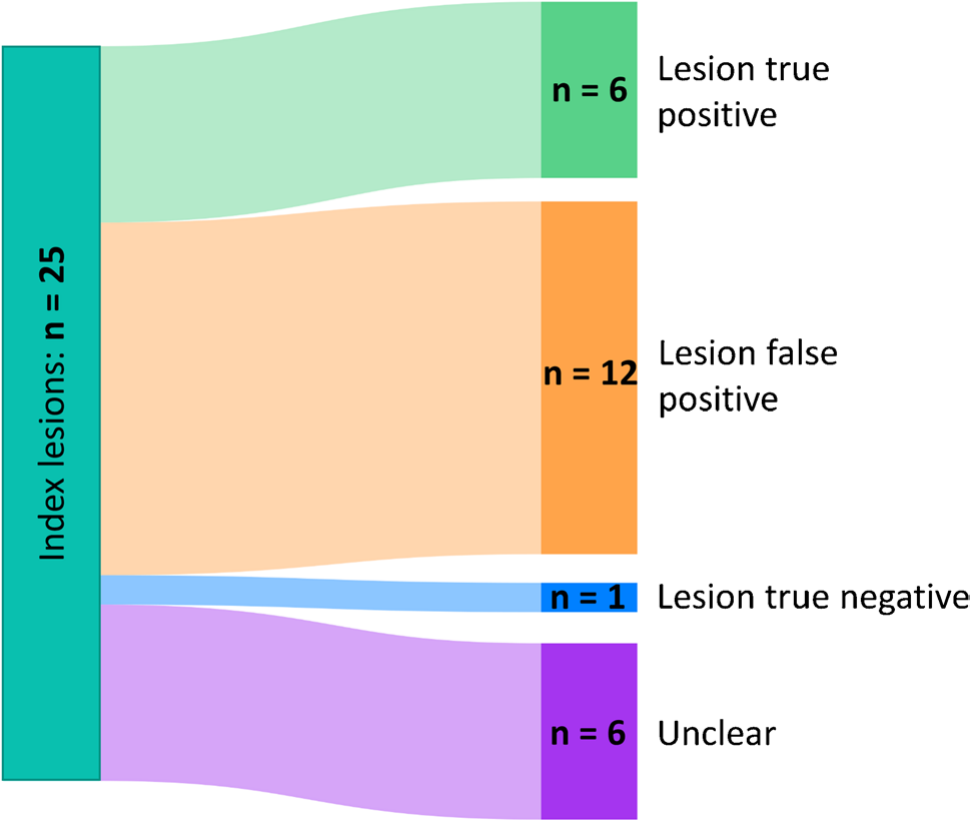
Sankey diagram of the lesion-based review of all index lesions

Clinical follow-up information is provided in Table 3. The preoperative and first follow-up PSA are illustrated in Fig. 1. Besides the first PSA after PSMA-RGS, no further information was available in patient 10 (lost to follow-up). The results of the review of all 25 index lesions are summarized in Table 3, Fig. 2, and Supplementary Table 1.

**Table 3:**
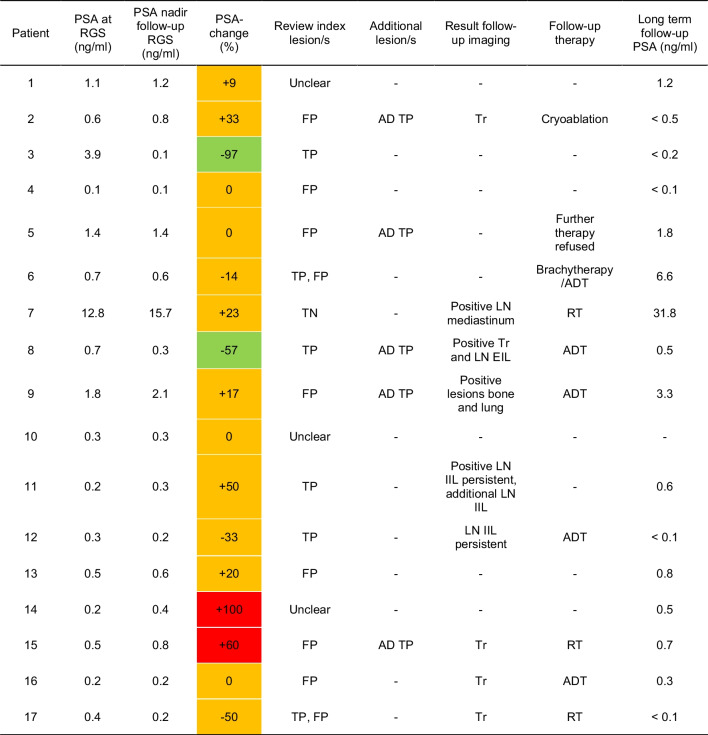
Clinical follow-up information

*ADT*, androgen deprivation therapy; *AD TP*, additional true positive; *EIL*, external iliac left; *FP*, false positive; *IIL*, internal iliac left; *LN*, lymph node; *PSA*, prostate-specific antigen; *RT*, radiotherapy; *TN*, true negative; *TP*, true positive; *Tr*, local recurrence

Green – PSA decrease >50%; yellow – PSA change ≤50%; red – PSA increase >50%

#### Lesion true positive

In patients 3 and 8, the PSMA-positive lesions were most probably true positive and successfully excised. The PSA of patient 3 decreased drastically from 3.9 to 0.1 ng/ml after PSMA-RGS and remained below 0.2 ng/ml until the last follow-up (Table 3, Fig. 1). In patient 8, the postoperative PSA sank from 0.7 to 0.3 ng/ml. Furthermore, the preoperatively identified PSMA-positive lymph node was not detectable on a follow-up PSMA PET/CT three months after PSMA-RGS. However, one PSMA-positive contralateral lymph node and two local recurrences (Supplementary Fig. 1) were not described in the initial report and were not addressed during surgery. All three lesions demonstrated a progressive PSMA expression on follow-up [^18^F]PSMA-1007 PET/CT. Patient 6 demonstrated a focal PSMA expression at the anastomosis which was also highly positive on the corresponding [^99m^Tc]Tc-PSMA-I&S SPECT/CT and therefore interpreted as true positive although the patient had received brachytherapy before salvage surgery (Supplementary Fig. 1). Due to an inadequate PSA response after the additional PSMA-RGS, an androgen deprivation therapy was started in patient 6. PSMA-positive lymph nodes of patients 11 and 12 were found in the same locations on follow-up PSMA PET as on the preoperative scans (Supplementary Fig. 1). Surgeries were complicated in both patients due to peritoneal scarring from previous interventions, leading to postoperative bleeding in patient 11 and an injury of the left ureter in patient 12 (Clavien-Dindo grade IIIb, respectively). Lastly, patient 17 demonstrated a PSMA-positive lesion at the level of anastomosis that was also evident on follow-up [^68^Ga]Ga-PSMA-11 PET five months after PSMA-RGS.

**Fig. 3:**
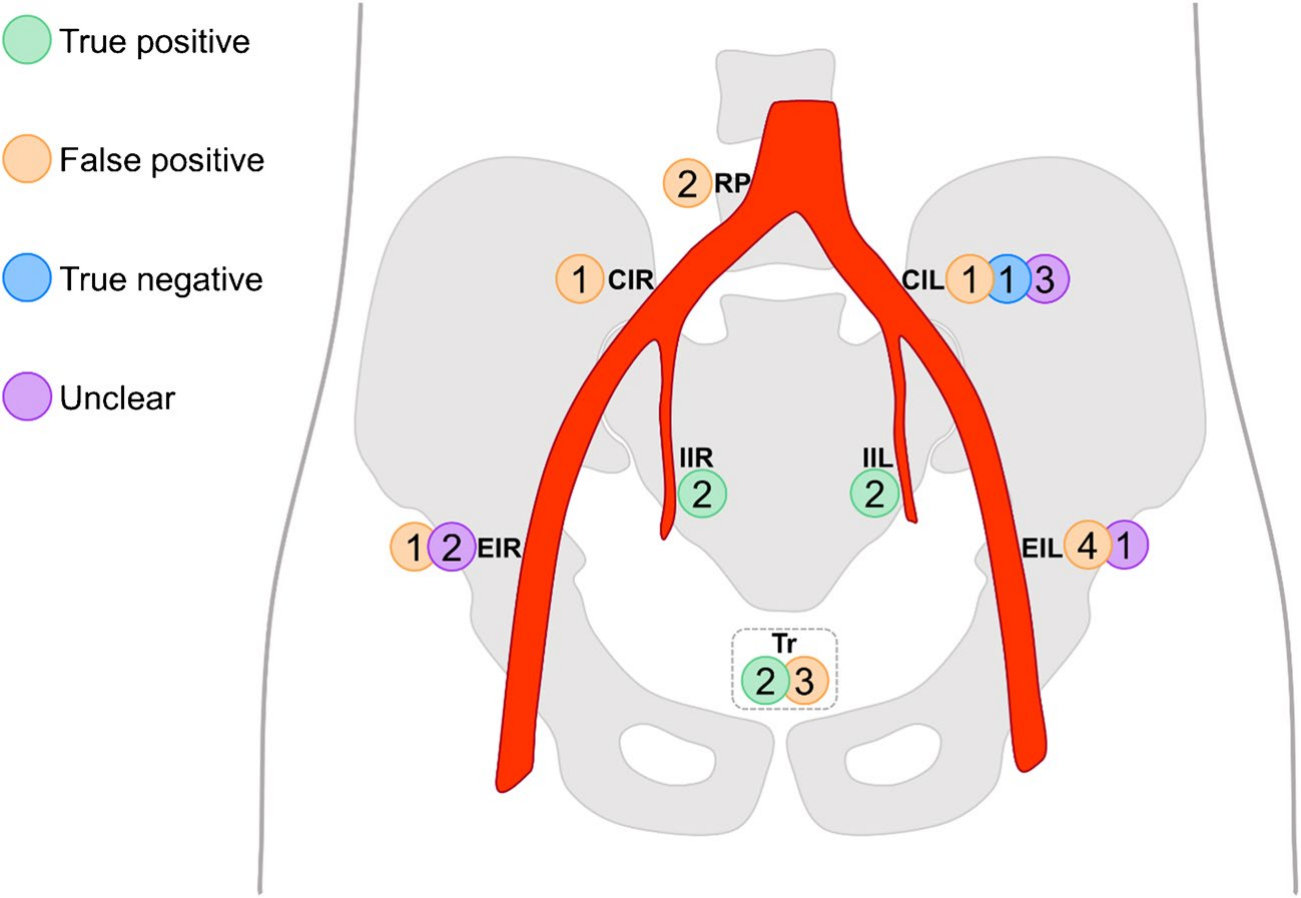
Index lesion distribution and status after retrospective analysis. The number of lesions that were assessed as true positive/false positive/true negative/additional true positive/unclear are provided in circles next to their respective locations. CIL = common iliac left, CIR = common iliac right, EIL = external iliac left, EIR = external iliac right, IIL = internal iliac left, IIR = internal iliac right, M1b = bone lesion, RP = retroperitoneal, Tr = local recurrence

All true positive index lesions were located either in the prostatic fossa or adjacent to the internal iliac arteries (Fig. 3). The median SUVmax of the true positive index lesions was 8.9 (IQR 3.8–13.8) with a median visual score of 2 (IQR 1–3).

#### Lesion false positive

After revision of the PSMA PET images and clinical follow-up, twelve index lesions of nine patients (2, 4–6, 9, 13, 15–17) were interpreted as false positives (Supplementary Table 1); 6 of these patients (2, 5, 6, 9, 15, 17) demonstrated additional PSMA-positive lesions besides the false-positive index lesions. As mentioned above, patients 6 and 17 also demonstrated index lesions compatible with local recurrences which were mentioned in the initial reports and treated by brachytherapy/external beam radiotherapy, respectively (Table 3). Patients 2, 5, and 15 exhibited focal PSMA expressions compatible with local recurrences that were not described in the initial reports. These foci were interpreted as the most probable causes of biochemical recurrence. In patient 9, a sclerotic bone lesion of the first rib on the left was already slightly PSMA positive on the preoperative PET/CT. It demonstrated an increased PSMA expression on follow-up imaging (Supplementary Fig. 1). Patient 4 had no additional PSMA-positive lesions besides the ones described in the initial reports. However, the PSA of patient 4 remained at values below 0.1 ng/ml 36 months after RGS without any further treatment during this time, rendering the initially described lesions false positive. In patient 13, a urine artifact was interpreted as a PSMA-positive lymph node which was located adjacent to the right ureter on a [^68^Ga]Ga-PSMA-I&T PET/CT. No other PSMA-positive lesions were found on this scan. The PSA of patient 13 increased slowly from 0.5 to 0.8 ng/ml 22 months after RGS without any further treatment. In patients 15, 16, and 17, the low PSMA-expressing false-positive index lesions were not identifiable on follow-up scans. A focal uptake at the anastomosis that was not visible on the preoperative scan was seen on a follow-up PSMA PET/CT of patient 16.

Three false-positive lesions were found in the prostatic fossa. Of these, two were diagnosed in patients who had received [^68^Ga]Ga-PSMA-I&T with high bladder activity (patients 9 and 16) and one case with low urinary excretion in a [^18^F]PSMA-1007 PET/CT (patient 4). All false-positive lymph nodes were either located adjacent to the distal external iliac arteries or outside the true pelvis (Fig. 3, Supplementary Fig. 1). None of these cases demonstrated other PSMA-expressing lymph nodes in regions that were closer to the prostatic fossa (i.e., internal iliac arteries, obturator, perirectal). The median SUVmax of the false-positive lesions was 2.5 (IQR 2.2–5) with a median visual score of 1 (IQR 1–1).

#### Lesion true negative

Patient 7 underwent PSMA-RGS, although no adequate PSMA-positive correlate for the biochemical recurrence (11 ng/ml) after RP was found on the preoperative PSMA PET. Only one slightly enlarged lymph node next to the left common iliac artery with low PSMA expression was described in the initial report, in which it was not seen as suspicious for PCa. No other PSMA-positive lesions were evident. The most probable reason for the PSA recurrence was identified on a follow-up [^68^Ga]Ga-PSMA-I&T PET 31 months after PSMA-RGS (PSA: 31 ng/ml). Here, a large PSMA-positive lymph node conglomerate was found in the lower mediastinum (Supplementary Fig. 1). The lymph node next to the left common iliac artery was not visible on follow-up imaging.

#### Unclear

Patients 1, 10, and 14 received no further diagnostic workup and no further therapies during follow-up. Moreover, no additional lesions compared to the initial reports were found on the preoperative PSMA PET scans, rendering these cases unclear. PSA values increased slowly in patients 1 and 14 after PSMA-RGS (patient 1: 1.1 ng/ml → 1.2 ng/ml after 12 months; patient 14: 0.2 ng/ml → 0.5 ng/ml after 23 months). Patient 10 was lost to follow-up, and no further PSA values after surgery were available.

All unclear lesions were located next to the external iliac arteries or the left common iliac artery (Fig. 3). The median SUVmax of the unclear index lesions was 4 (IQR 3.2–5.4) with a median visual score of 1 (IQR 1–1).

## Discussion

The emergence of PSMA-targeted imaging revolutionized PCa diagnostics and paved the way for new innovative treatment strategies. On this basis, PSMA-radioguidance was able to improve the detection rate of PCa lesions during salvage surgery, reducing negative histopathological results compared to conventional salvage lymphadenectomy [28]. In a recent analysis, Knipper et al. were able to remove metastatic tissue in almost 95% of patients using PSMA-RGS [24]. Similarly, PSMA-radioguidance helped to identify PCa metastases in most patients of the presented cohort. However, the histopathological analysis was negative in 5.6% of cases. Here, we demonstrate that the most frequent reason for negative histopathology in the analyzed group was false-positive lesions on preoperative PSMA PET. Metastases were missed by surgery or histopathology only in a small number of cases.

Most PCa upregulate PSMA. However, it is also expressed in multiple benign tissues and within the neovasculature of other solid malignancies [29, 30]. Unspecific foci are frequently observed on PSMA imaging, especially in ^18^F-labeled radioligands [31–33]. This underlines the importance of reader experience and adequate morphological imaging to identify unspecific uptake, which can be assumed in the encountered false-positive lesions. The common denominator of these lesions was their low PSMA positivity (median visual score 1). However, PSMA expression alone is not sufficient to assess a lesion. While the probability of PCa is certainly higher with increasing PSMA positivity, lesions with low uptake can also represent PCa manifestations (e.g., patients 6, 11, 17; Supplementary Fig. 1). The presented analysis suggests that the lesion site may be another indicator to evaluate the probability of a PCa recurrence. Only a few false-positive lesions were described in the prostatic fossa, including two suspected local recurrences in patients with high urine activity, impeding PET interpretation. All false-positive lymph nodes were located either at the level of the distal external iliac arteries or outside the true pelvis. None of these cases demonstrated positive lymph nodes in the internal iliac, obturator, or perirectal regions. Consequently, low uptake of a pelvic lymph node that is distant to the prostatic fossa or a lymph node outside the true pelvis, without evidence of lymph nodes closer to the fossa, should only be considered as suspicious with caution. For example, this could be the case after radiotherapy to the pelvic lymphatics. In these patients, disease recurrence may present in distant locations without evidence of PCa closer to the prostatic fossa.

Interestingly, true positive lesions were also identified in the presented cohort of patients without evidence of PCa cells on histopathology of specimens from salvage lymph node dissection. In two cases, the respective lesions were not reached by PSMA-RGS. Both surgeries were complicated by scarring after previous interventions, and the respective lesions lay deep in the regions of the internal iliac arteries, which are technically challenging to reach. Similarly, Rauscher et al. described a missed lymph node at the same site [18]. In another investigation of patients who underwent salvage lymph node dissection without PSMA-RGS, the internal iliac region was also more often affected by disease persistence than other locations [34], indicating that the risk for a negative outcome is increased in internal iliac lymph nodes. However, compared to the entire study population of 302 patients, missing a highly PSMA-positive lesion is a rare event in PSMA-RGS (<1%). On the contrary, the presented results also show that negative histopathology does not automatically imply an unsuccessful surgery. Two PSMA-expressing lymph nodes of two other patients were also seen as true positive by clinical/imaging follow-up, although the histopathological workup did not yield PCa cells. In patient 3, the postoperative PSA decreased by over 95%, corresponding to a complete biochemical response. In patient 8, the PSA decreased by over 50% after PSMA-RGS and the respective lymph node was not detectable on a follow-up PSMA PET/CT. Reasons for the negative pathologies in both cases can only be speculated. Either the specimens were not correctly transferred to the pathology department, or the lymph nodes were not found within the tissue samples. While the latter seems unlikely in the case of patient 3 (lesion diameters 9 × 18 mm), it may be an explanation in patient 8 (lesion diameters 3 × 6 mm).

PSMA-RGS was conducted in one patient (patient 7) with a low PSMA-expressing lymph node on the preoperative [^18^F]PSMA-1007 PET/CT, which was not seen as an adequate correlate of the highly elevated PSA after prostatectomy (11 ng/ml). Previous reports stated that PSMA-RGS could identify more PSMA-positive lesions than the corresponding preoperative PET/CT scans [18, 20, 35], probably due to the limited sensitivity and spatial resolution of PET imaging. Nevertheless, the presented case supports that PSMA-RGS is not promising if the clinical context and PSMA imaging are not compatible. As confirmed on a follow-up scan, the most probable location of the disease recurrence was not the above-mentioned iliac lymph node but rather a conglomerate of mediastinal lymph nodes that exhibited only an unspecific radioligand uptake at the time of the initial scan (Supplementary Fig. 1).

Lastly, no reliable reason for the negative histopathological results of three cases was found, including one patient who was lost to follow-up. The available PSA values increased slowly in the other two patients. Both were asymptomatic until the last follow-up which is why no further diagnostic tests were initiated. However, these cases lead to the most important limitations of this study: its retrospective design and the highly selected patient cohort. Although the underlying database was maintained prospectively, it did not offer the benefits of a prospective trial with planned follow-up PSMA PET to review outcomes. Furthermore, PSMA-RGS was only performed in patients who were found to be eligible for surgery by the treating physician, introducing a selection bias in the primary cohort as well as the presented subgroup. Next, only cases with a negative histopathology were included. Lesion distribution of positive cases was therefore not analyzed, limiting the presented results. Furthermore, PSMA-radioguidance reduced the number of negative histopathological examinations drastically compared to the literature, inevitably leading to the small sample size of the presented cohort, which may not comprise all reasons for a negative outcome. However, it represents the largest analysis of this very specific group, offering valuable insights into the limitations of PSMA imaging and salvage surgery. The employed radioligands and PET scanners differed between patients and follow-up studies, impairing the comparability of quantitative and semiquantitative parameters. To mitigate this, lesions were evaluated qualitatively based on the PROMISE V2 criteria. Moreover, it must be assumed that the expertise of the respective centers differed. Reader experience is highly important in the interpretation of PSMA-targeted imaging, especially if lesion uptake is low. Centralized image interpretation or consensus reads could help to improve the specificity of PSMA PET interpretation. Lastly, follow-up PSMA PET was not available in all cases because the indication for imaging was based on the clinical needs of each individual patient. Again, a prospective trial with planned follow-up PSMA-targeted imaging at a set interval after PSMA-RGS would help to interpret cases and identify important pitfalls.

## Conclusion

In most cases with a negative histopathological result of the resected specimens from PSMA-RGS, the identified target lesions were false positive on the preoperative PSMA PET. Unspecific uptake should be considered in low PSMA-expressing lymph nodes next to the distal external iliac arteries or outside the pelvis, especially if no other PSMA-positive lymph nodes closer to the prostatic fossa are evident. Rarely, metastases were not successfully reached during surgery or missed in the histopathological workup.

### Supplementary Information

Below is the link to the electronic supplementary material.

Supplementary file1 (PDF 4533 KB)

Supplementary file2 (DOCX 26 KB)

## Data Availability

The datasets generated during and/or analyzed during the current study are available from the corresponding author upon reasonable request.

## Abbreviations

IQR: interquartile range
PCa: prostate cancer
PSA: prostate-specific antigen
PSMA: prostate-specific membrane antigen
RGS: radioguided surgery
