# Supplementary material for: Prostate-specific membrane antigen radioguided surgery with negative histopathology: an in-depth analysis
Source: Eur J Nucl Med Mol Imaging. 2023 Sep 26;51(2):548–57. doi: 10.1007/s00259-023-06442-7 (PMC10774205; doi:10.1007/s00259-023-06442-7)
Supplement: Supplementary file 1 — Supplementary file1 (PDF 4533 KB) [file 259_2023_6442_MOESM1_ESM.pdf]

Pat. Nr. 1
Lesion 1 (CIL)

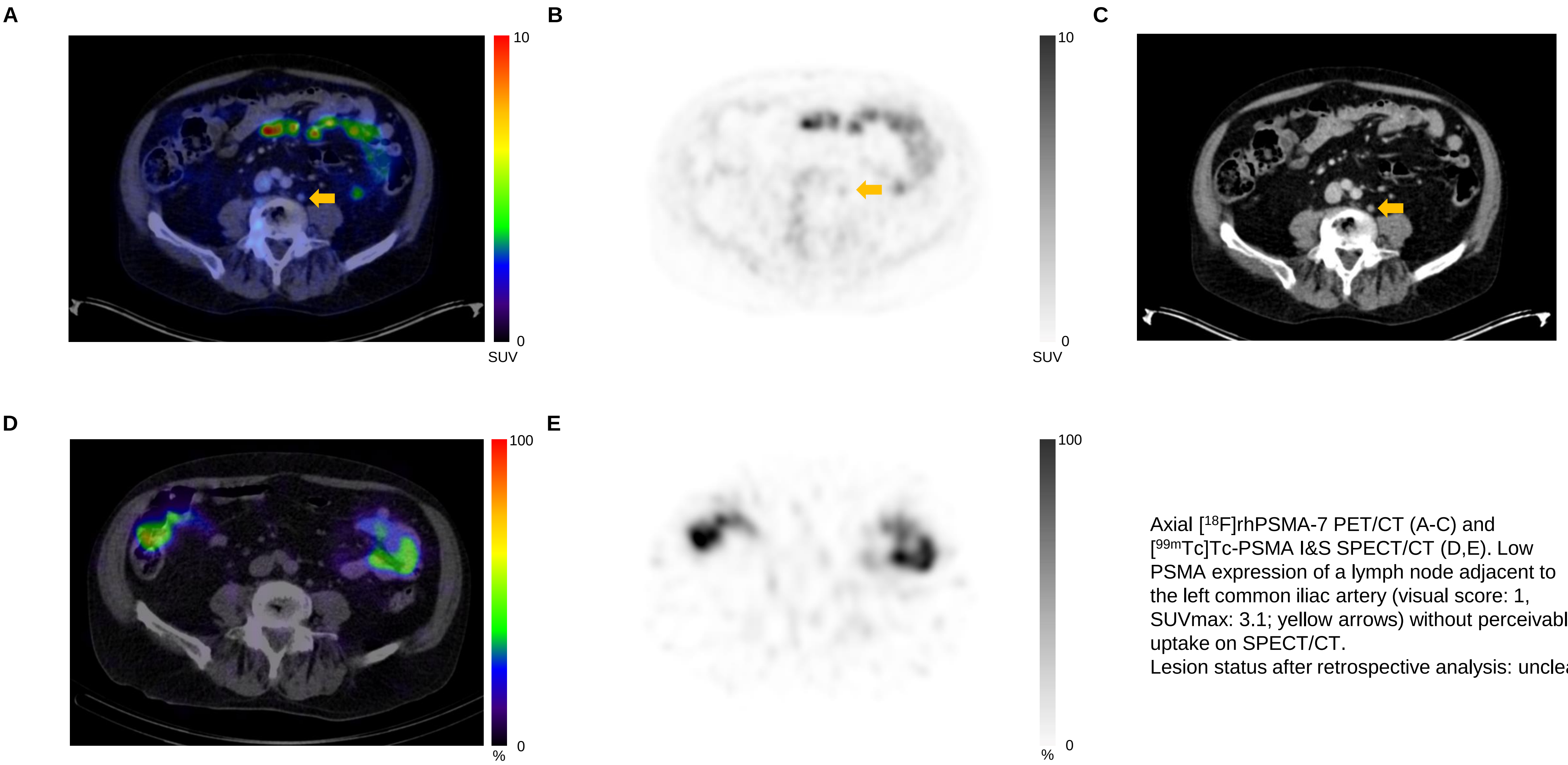

Axial [^{18}F]rhPSMA-7 PET/CT (A-C) and [^{99m}Tc]Tc-PSMA I&S SPECT/CT (D,E). Low PSMA expression of a lymph node adjacent to the left common iliac artery (visual score: 1, SUVmax: 3.1; yellow arrows) without perceivable uptake on SPECT/CT.
Lesion status after retrospective analysis: unclear

Pat. Nr. 1
Lesion 2 (EIL)

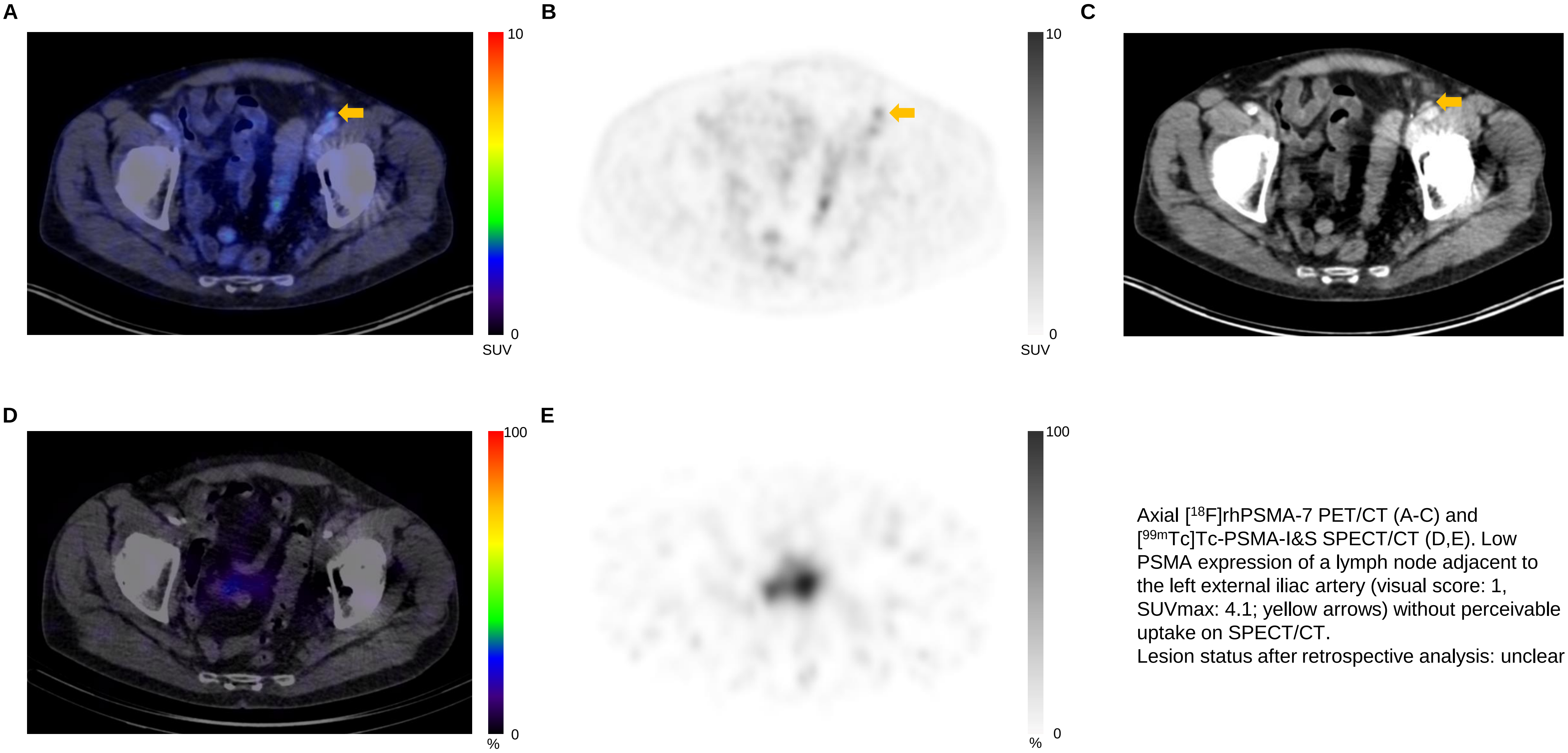

Axial [^{18}F]rhPSMA-7 PET/CT (A-C) and [^{99m}Tc]Tc-PSMA-I&S SPECT/CT (D,E). Low PSMA expression of a lymph node adjacent to the left external iliac artery (visual score: 1, SUVmax: 4.1; yellow arrows) without perceivable uptake on SPECT/CT.
Lesion status after retrospective analysis: unclear

Pat. Nr. 2
Lesion 1 (RP_1)

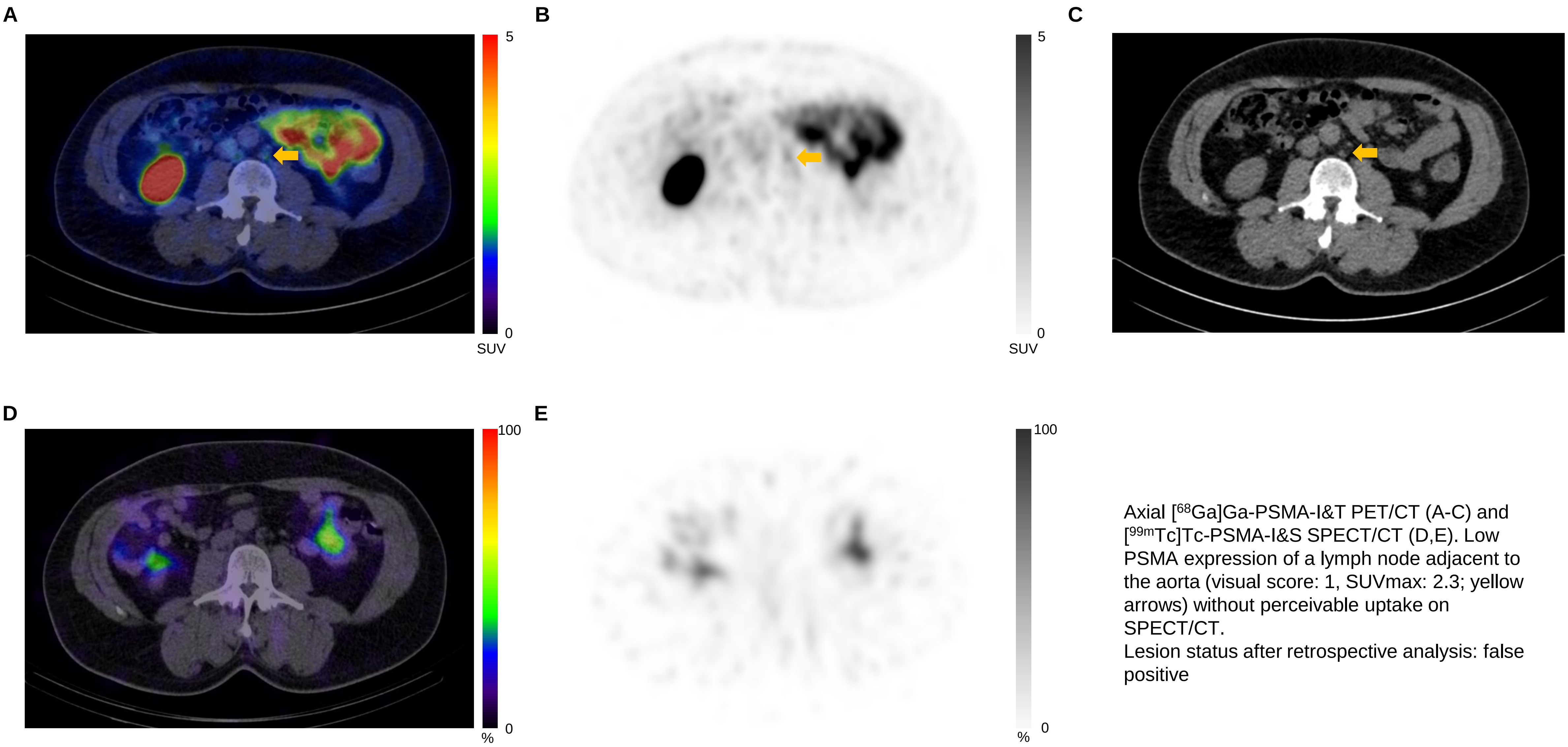

Axial [^{68}Ga]Ga-PSMA-I&T PET/CT (A-C) and [^{99m}Tc]Tc-PSMA-I&S SPECT/CT (D,E). Low PSMA expression of a lymph node adjacent to the aorta (visual score: 1, SUVmax: 2.3; yellow arrows) without perceivable uptake on SPECT/CT.
Lesion status after retrospective analysis: false positive

Pat. Nr. 2
Lesion 2 (RP_2)

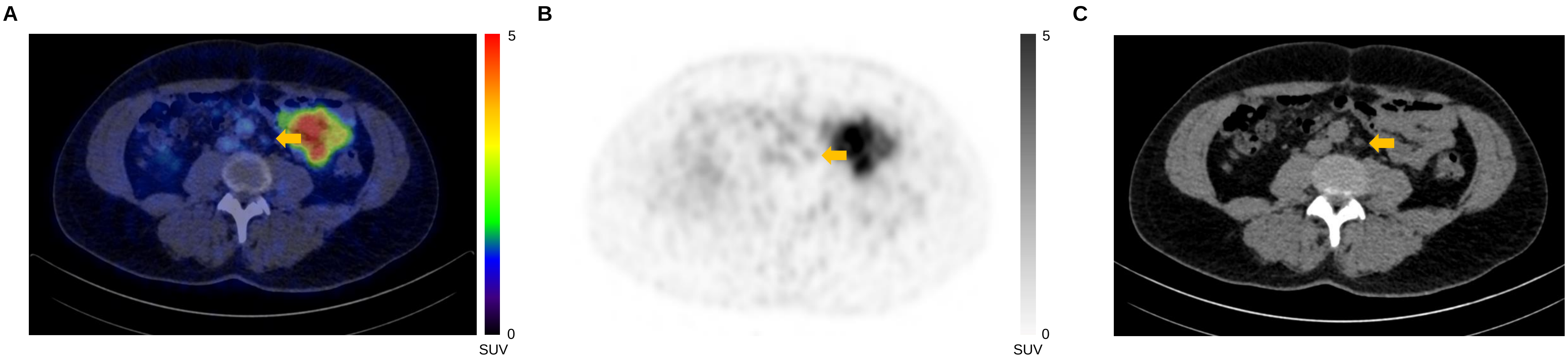

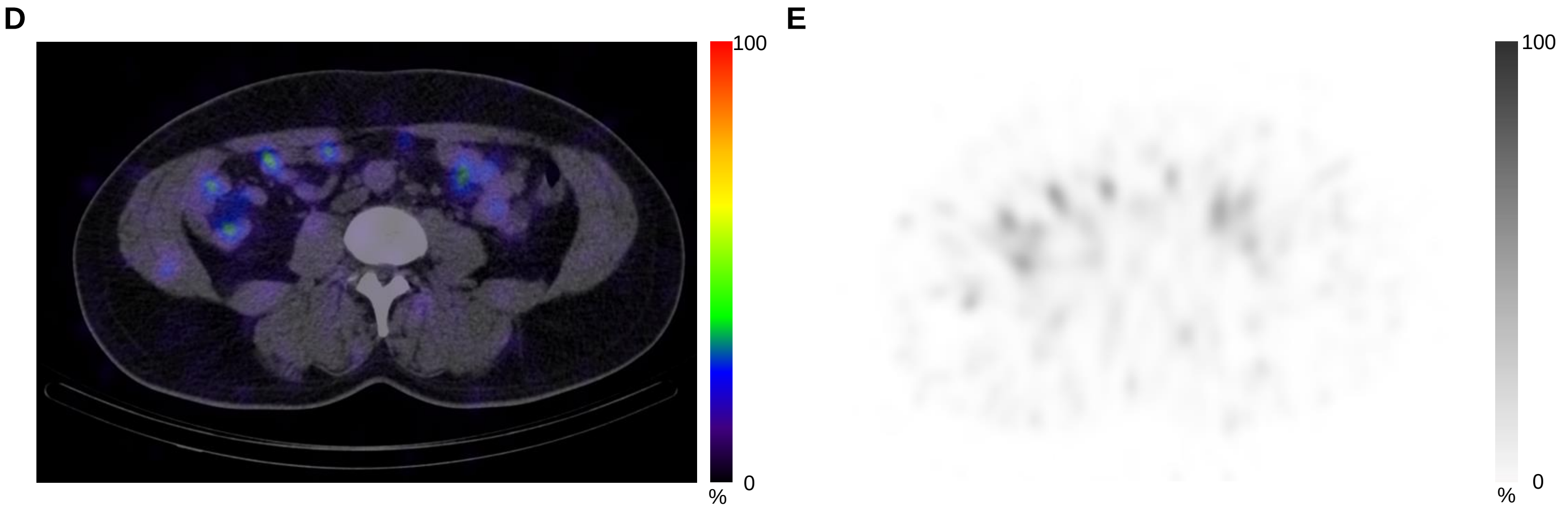

Axial [^{68}Ga]Ga-PSMA-I&T PET/CT (A-C) and [^{99m}Tc]Tc-PSMA-I&S SPECT/CT (D,E). Low PSMA expression of a second lymph node adjacent to the distal aorta (visual score: 1, SUVmax: 1.6; yellow arrows) without perceivable uptake on SPECT/CT.
Lesion status after retrospective analysis: false positive

Pat. Nr. 2

Tr (additional lesion)

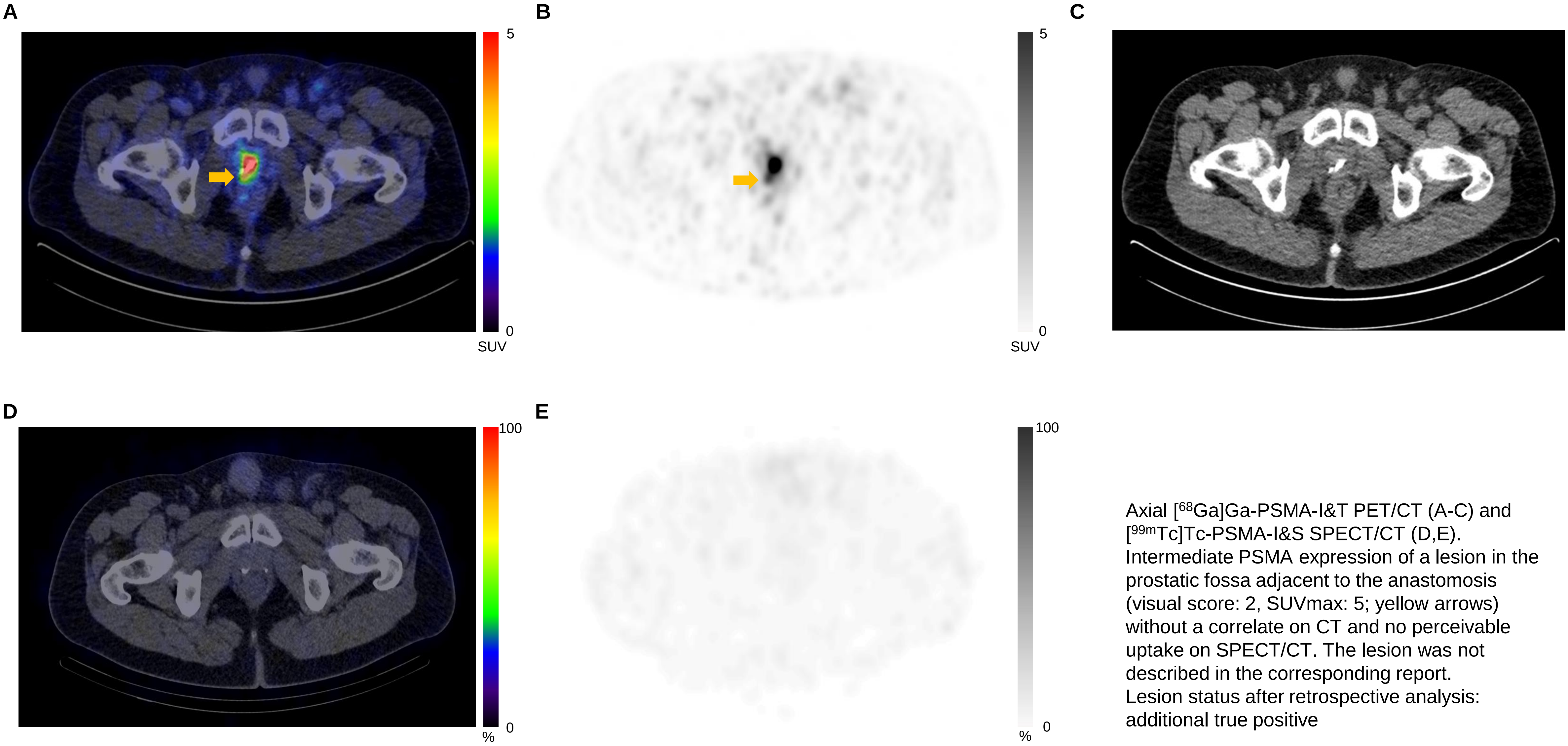

Axial [^{68}Ga]Ga-PSMA-I&T PET/CT (A-C) and [^{99m}Tc]Tc-PSMA-I&S SPECT/CT (D,E). Intermediate PSMA expression of a lesion in the prostatic fossa adjacent to the anastomosis (visual score: 2, SUVmax: 5; yellow arrows) without a correlate on CT and no perceivable uptake on SPECT/CT. The lesion was not described in the corresponding report. Lesion status after retrospective analysis: additional true positive

Pat. Nr. 2

Tr (additional lesion, follow-up)

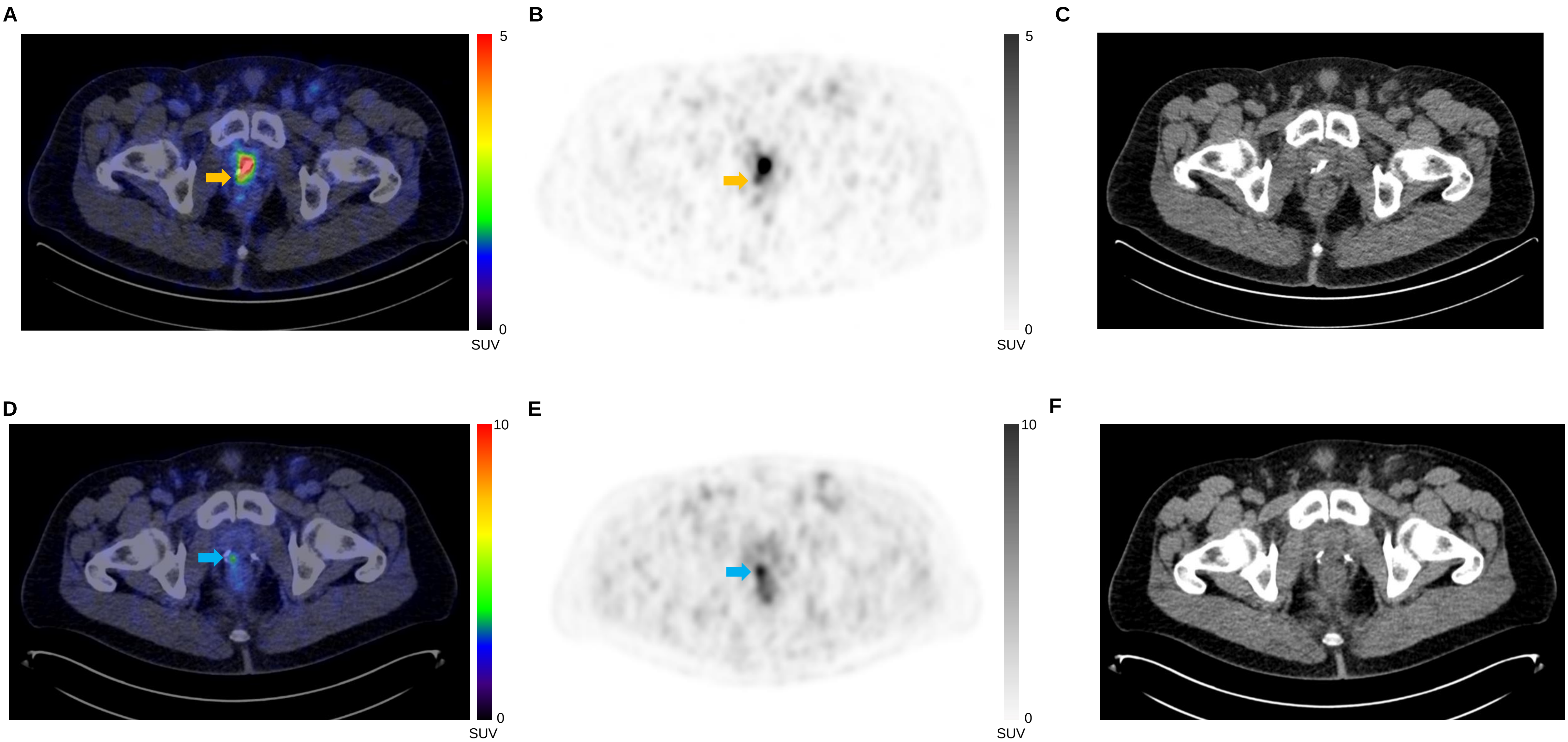

Preoperative [^{68}Ga]Ga-PSMA-I&T PET/CT (A-C) and follow-up [^{18}F]PSMA-1007 PET/CT 4 months later (D-F). Intermediate PSMA expression of a lesion in the prostatic fossa adjacent to the anastomosis on the preoperative scan (visual score: 2, SUVmax: 5; yellow arrows) with persistence on the follow-up PET/CT (visual score: 1, SUVmax: 4.6; blue arrows). Lesion status after retrospective analysis: additional true positive

Pat. Nr. 3
Lesion 1 (IIR)

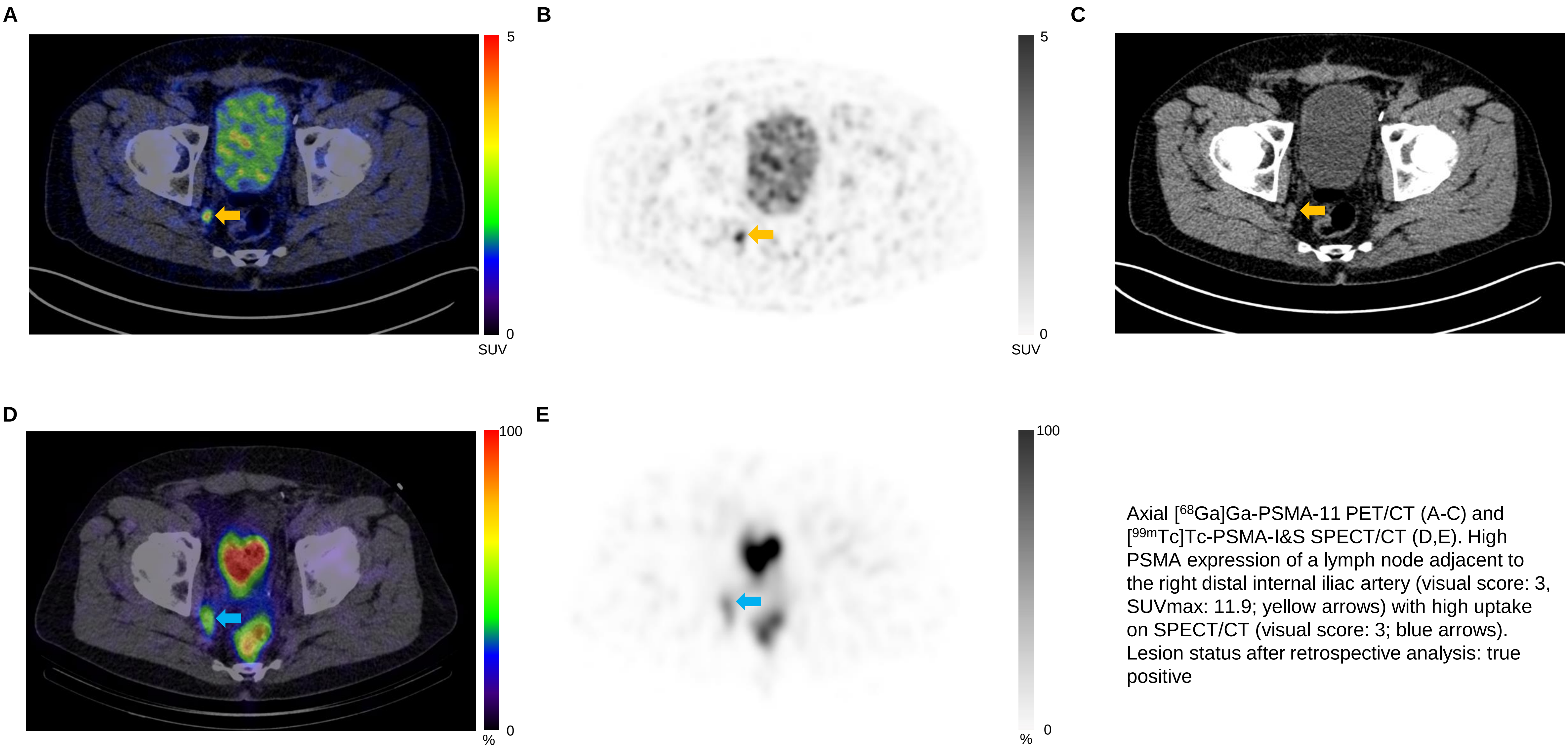

Axial [^{68}Ga]Ga-PSMA-11 PET/CT (A-C) and [^{99m}Tc]Tc-PSMA-I&S SPECT/CT (D,E). High PSMA expression of a lymph node adjacent to the right distal internal iliac artery (visual score: 3, SUVmax: 11.9; yellow arrows) with high uptake on SPECT/CT (visual score: 3; blue arrows). Lesion status after retrospective analysis: true positive

Pat. Nr. 4
Lesion 1 (EIL)

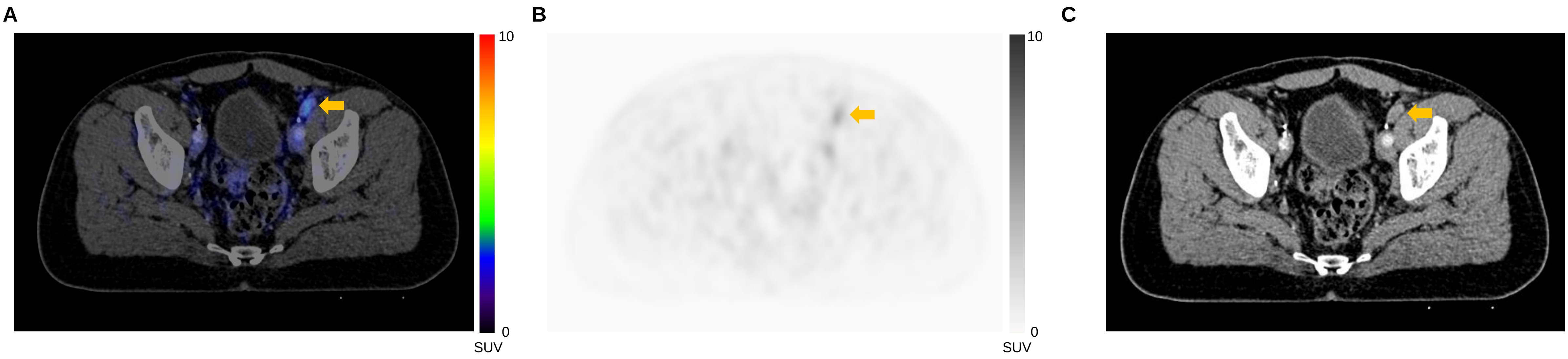

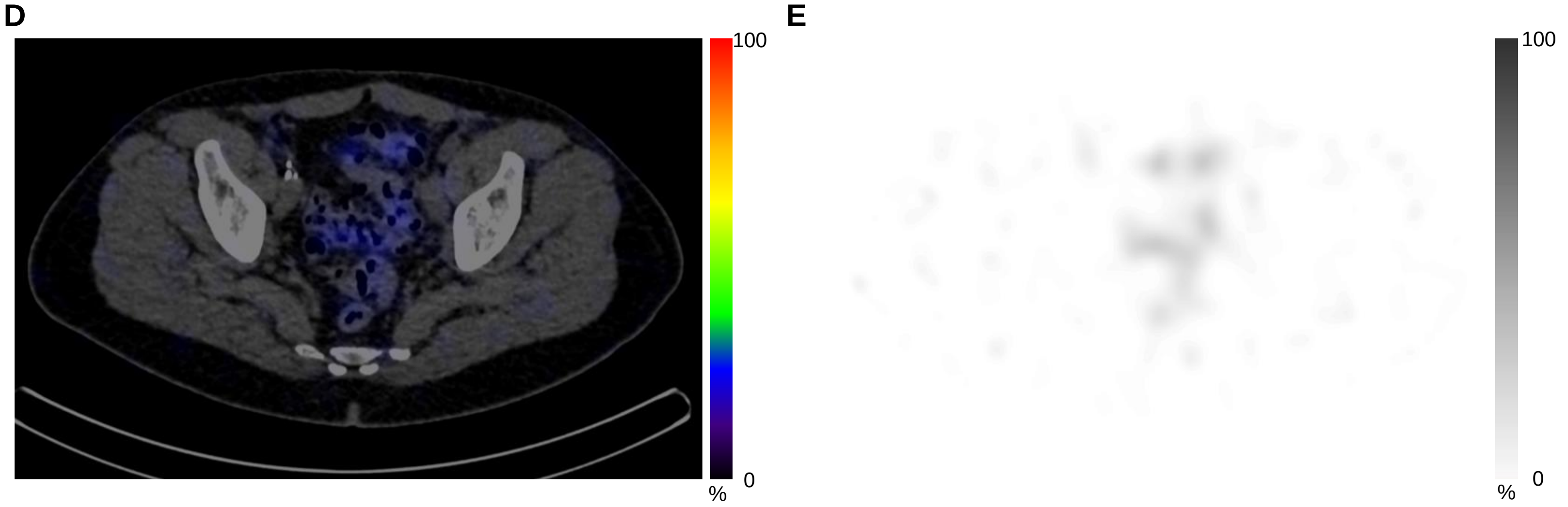

Axial [^{18}F]PSMA-1007 PET/CT (A-C) and [^{99m}Tc]Tc-PSMA-I&S SPECT/CT (D,E). Low PSMA expression of a lymph node adjacent to the left external iliac artery (visual score: 1, SUVmax: 3.9; yellow arrows) without perceivable uptake on SPECT/CT.
Lesion status after retrospective analysis: false positive

Pat. Nr. 4
Lesion 2 (Tr)

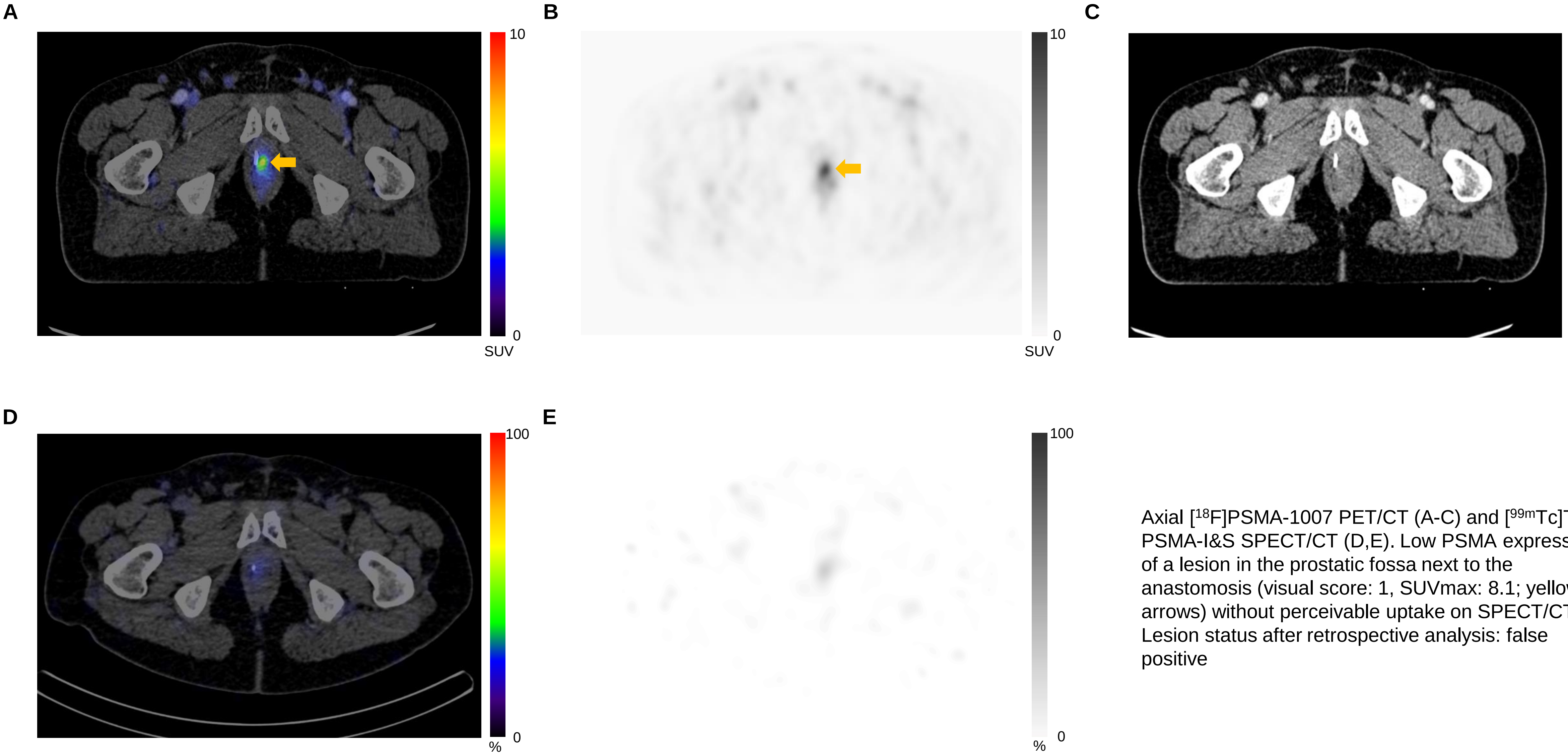

Axial [^{18}F]PSMA-1007 PET/CT (A-C) and [^{99m}Tc]Tc-PSMA-I&S SPECT/CT (D,E). Low PSMA expression of a lesion in the prostatic fossa next to the anastomosis (visual score: 1, SUVmax: 8.1; yellow arrows) without perceivable uptake on SPECT/CT. Lesion status after retrospective analysis: false positive

Pat. Nr. 5
Lesion 1 (EIL_1)

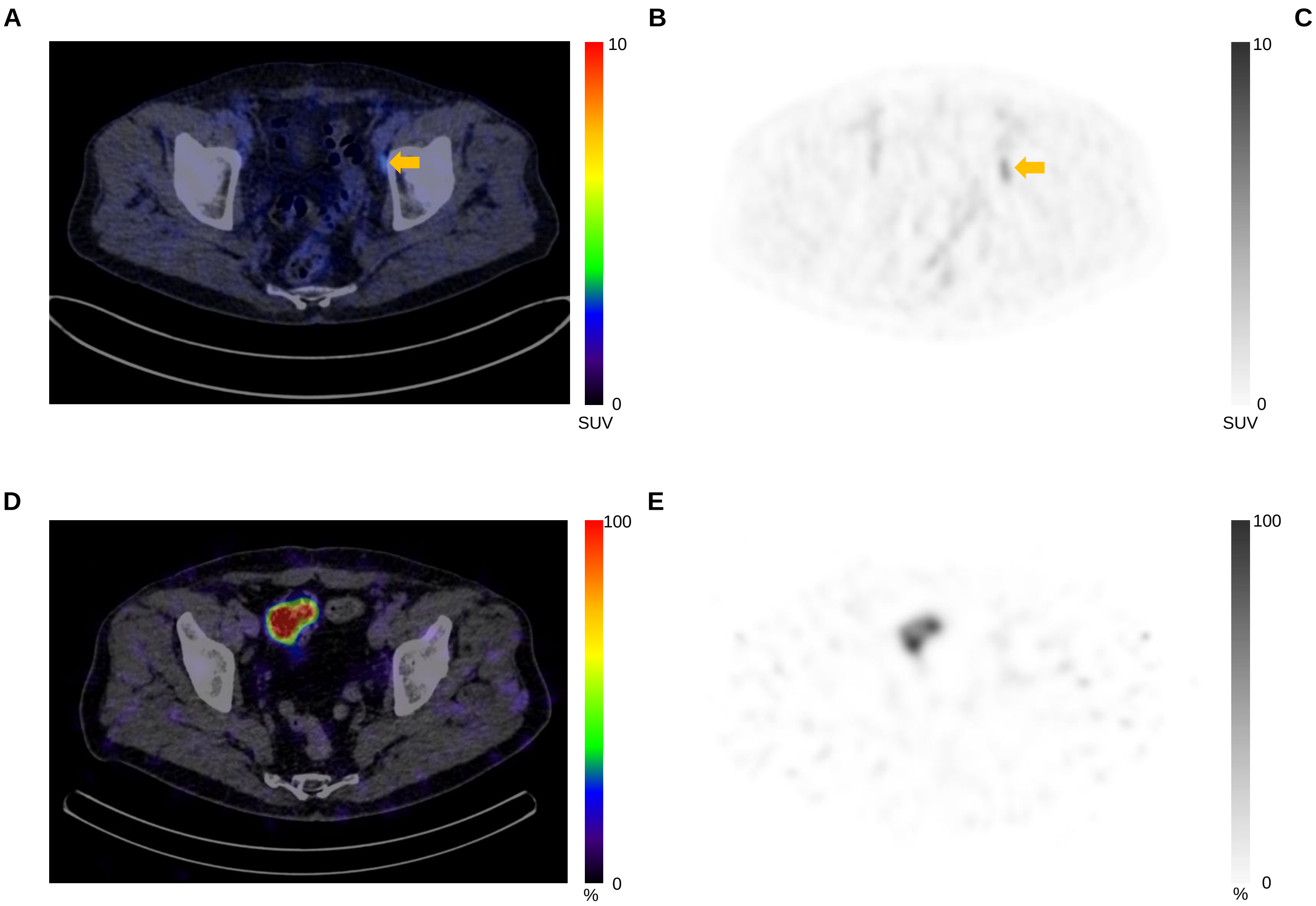

Axial [^{18}F]PSMA-1007 PET/CT (A-C) and [^{99m}Tc]Tc-PSMA-I&S SPECT/CT (D,E). Low PSMA expression of a lymph node adjacent to the left external iliac vessels (visual score: 1, SUVmax: 4.3; yellow arrows) without perceivable uptake on SPECT/CT.
Lesion status after retrospective analysis: false positive

Pat. Nr. 5
Lesion 2 (EIL_2)

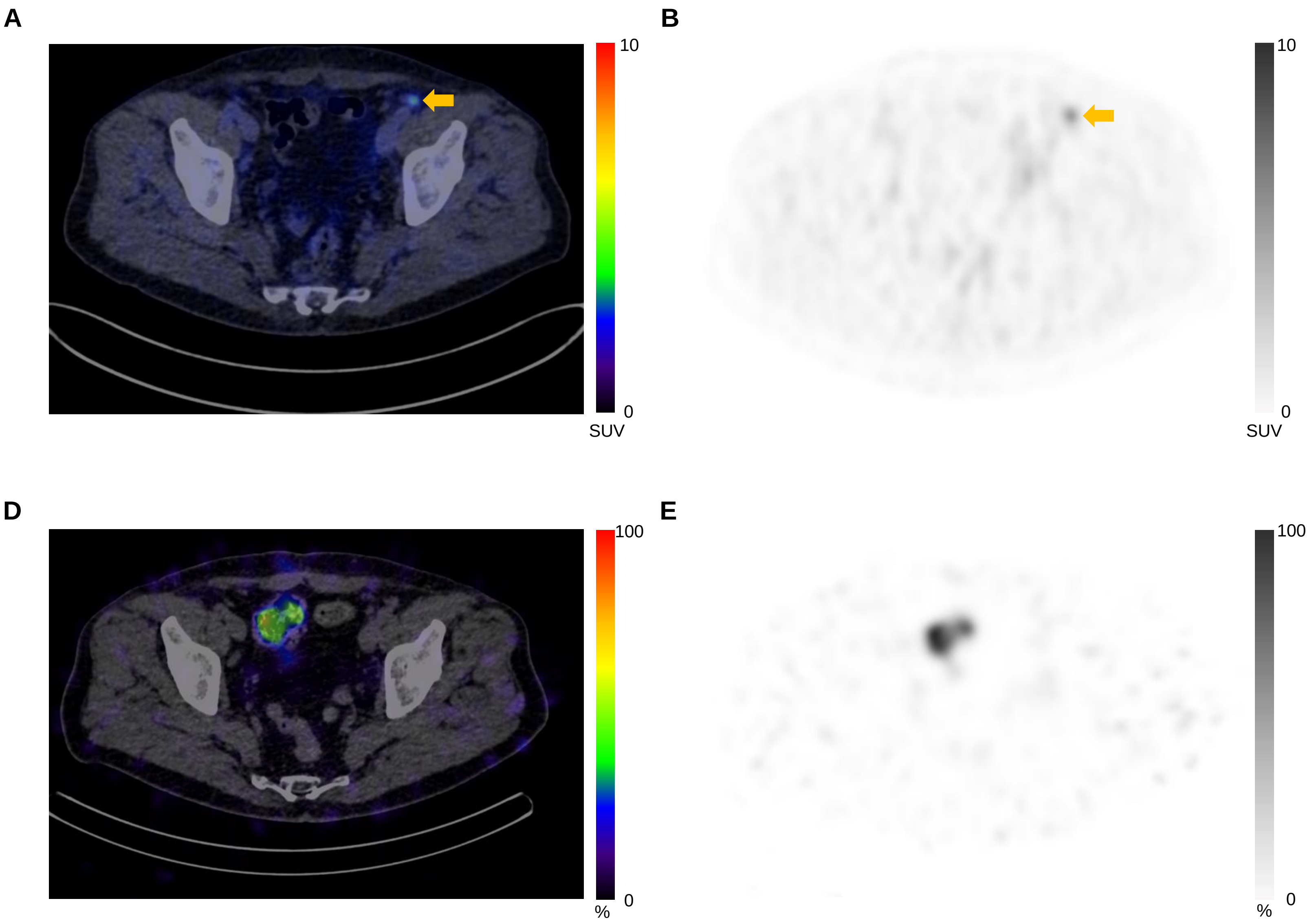

Axial [^{18}F]PSMA-1007 PET/CT (A-C) and [^{99m}Tc]Tc-PSMA-I&S SPECT/CT (D,E). Low PSMA expression of a lymph node adjacent to the left external iliac artery (visual score: 1, SUVmax: 5; yellow arrows) without perceivable uptake on SPECT/CT.
Lesion status after retrospective analysis: false positive

Pat. Nr. 5

Tr (additional lesion)

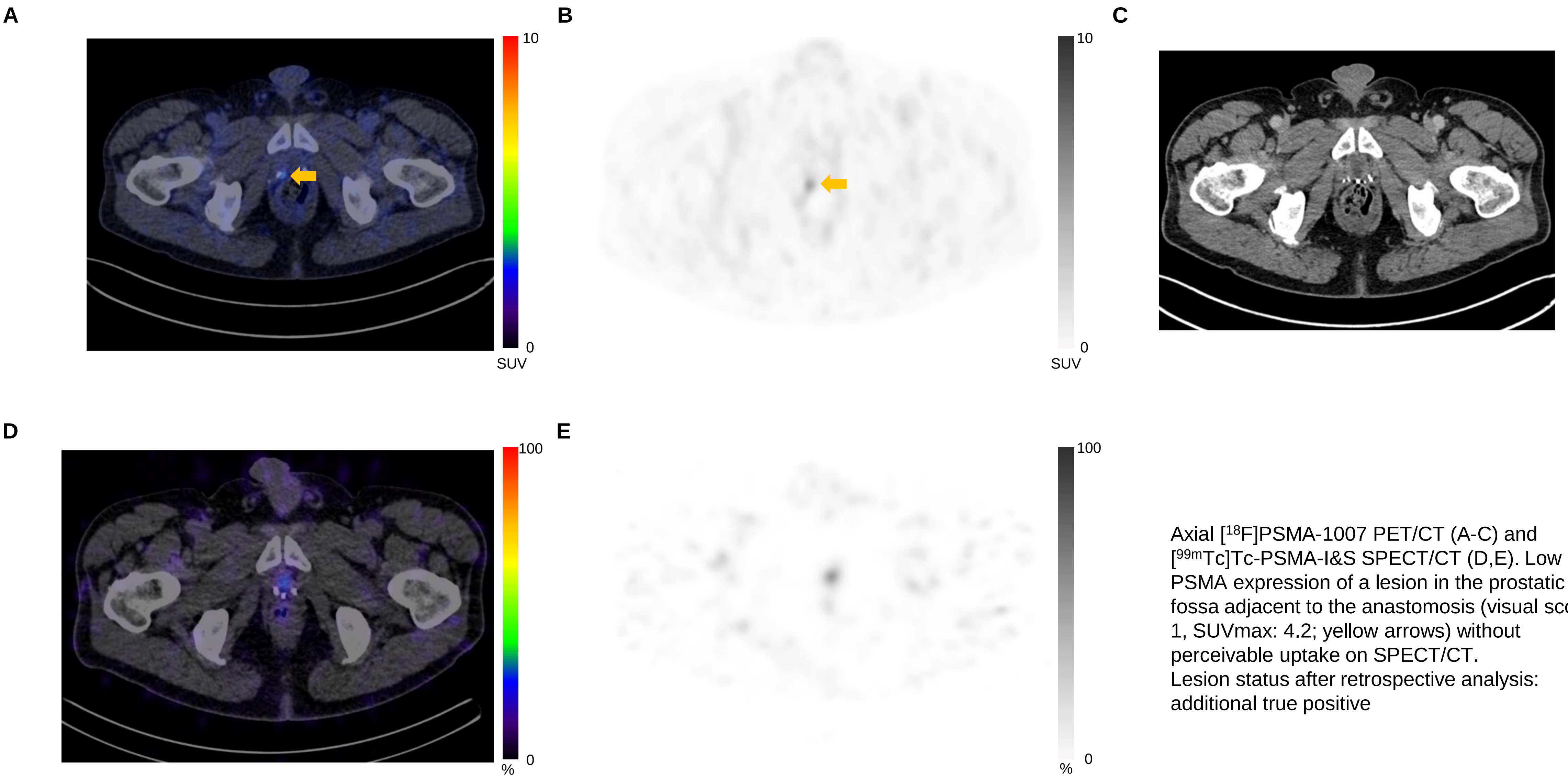

Axial [^{18}F]PSMA-1007 PET/CT (A-C) and [^{99m}Tc]Tc-PSMA-I&S SPECT/CT (D,E). Low PSMA expression of a lesion in the prostatic fossa adjacent to the anastomosis (visual score: 1, SUVmax: 4.2; yellow arrows) without perceivable uptake on SPECT/CT.
Lesion status after retrospective analysis: additional true positive

Pat. Nr. 6
Lesion 1 (Tr)

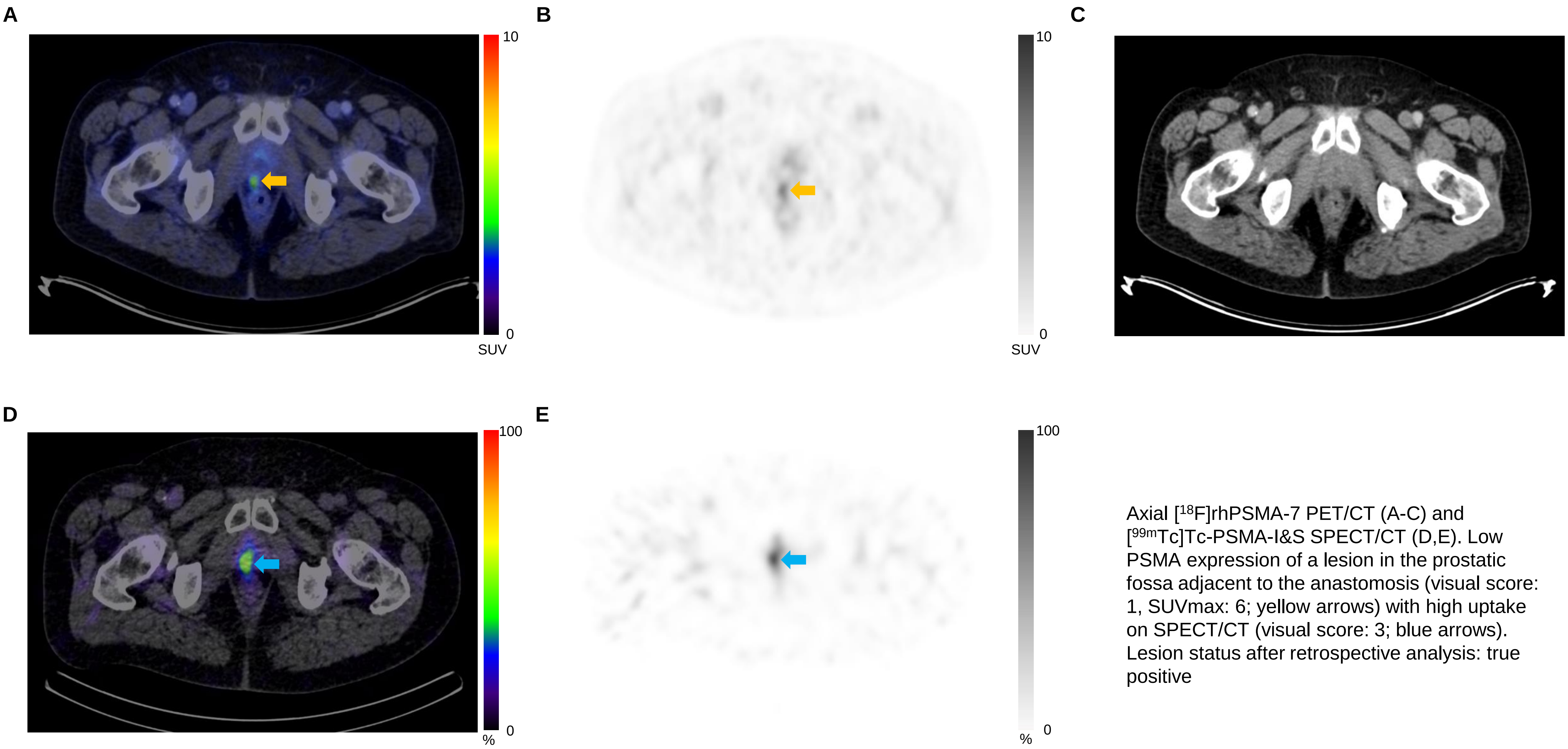

Axial [^{18}F]rhPSMA-7 PET/CT (A-C) and [^{99m}Tc]Tc-PSMA-I&S SPECT/CT (D,E). Low PSMA expression of a lesion in the prostatic fossa adjacent to the anastomosis (visual score: 1, SUVmax: 6; yellow arrows) with high uptake on SPECT/CT (visual score: 3; blue arrows). Lesion status after retrospective analysis: true positive

Pat. Nr. 6
Lesion 2 (EIR)

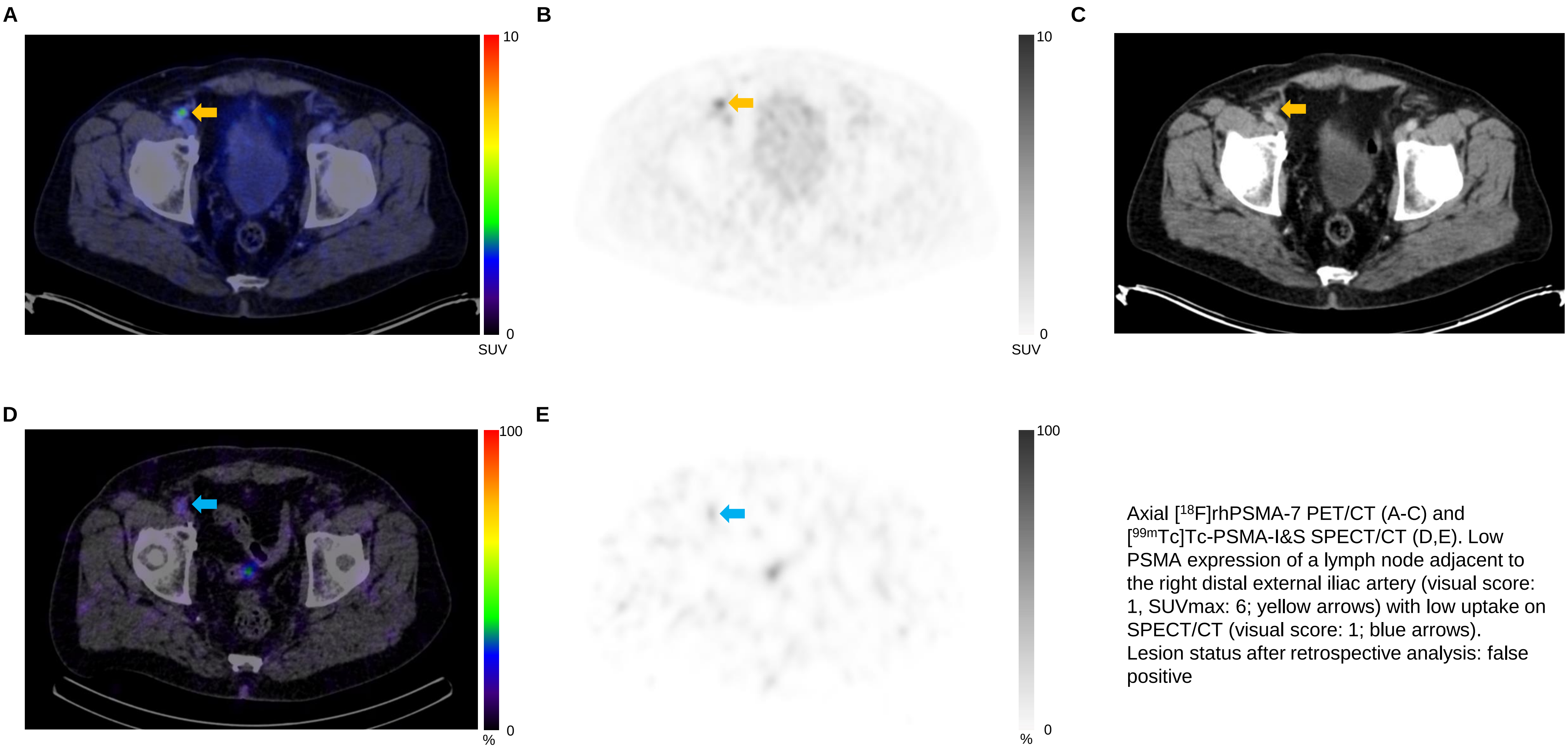

Axial [^{18}F]rhPSMA-7 PET/CT (A-C) and [^{99m}Tc]Tc-PSMA-I&S SPECT/CT (D,E). Low PSMA expression of a lymph node adjacent to the right distal external iliac artery (visual score: 1, SUVmax: 6; yellow arrows) with low uptake on SPECT/CT (visual score: 1; blue arrows). Lesion status after retrospective analysis: false positive

Pat. Nr. 7
Lesion 1 (CIL)

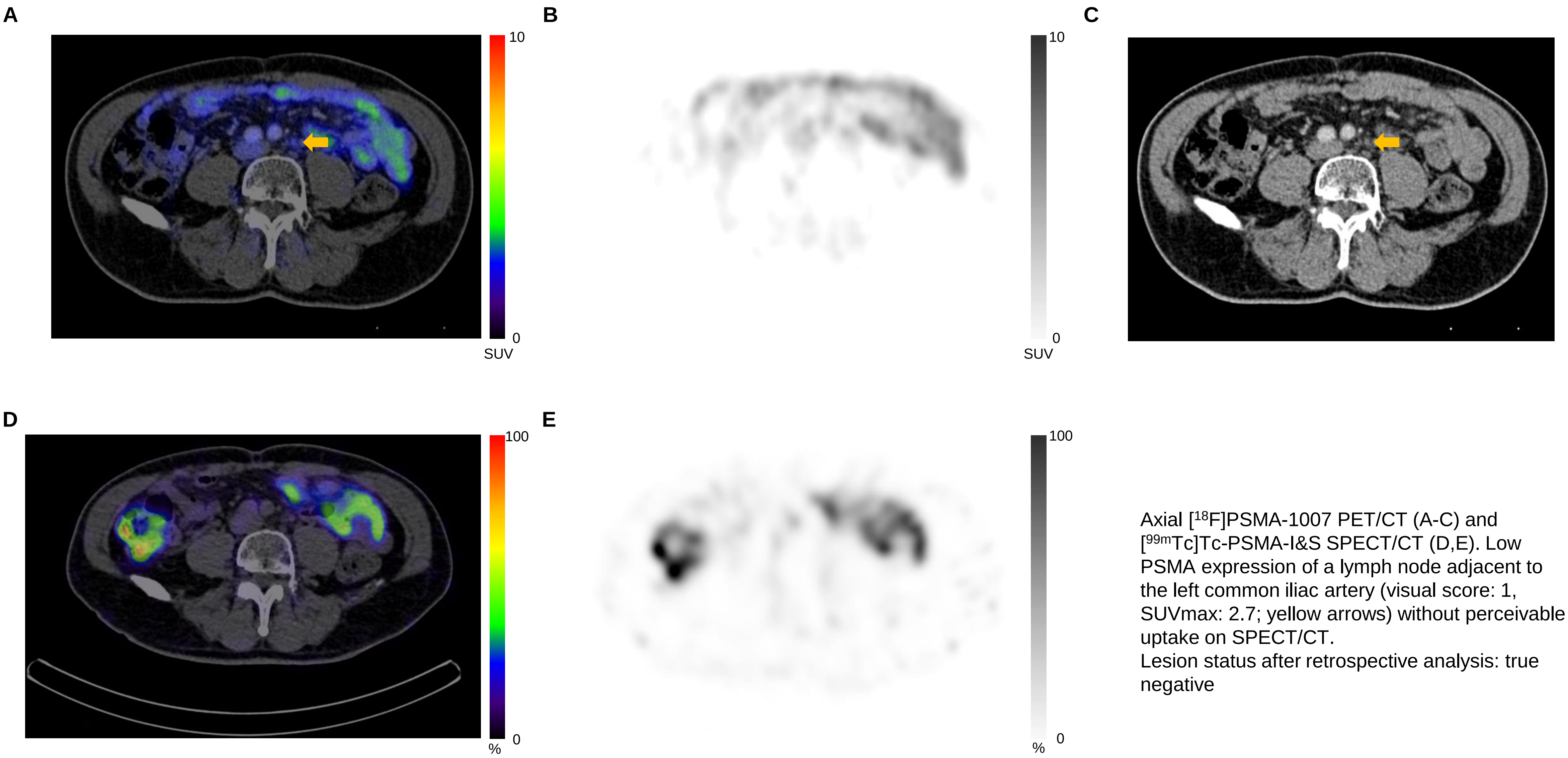

Axial [^{18}F]PSMA-1007 PET/CT (A-C) and [^{99m}Tc]Tc-PSMA-I&S SPECT/CT (D,E). Low PSMA expression of a lymph node adjacent to the left common iliac artery (visual score: 1, SUVmax: 2.7; yellow arrows) without perceivable uptake on SPECT/CT.
Lesion status after retrospective analysis: true negative

Pat. Nr. 7

Supradiaphragmatic lymph nodes on follow-up

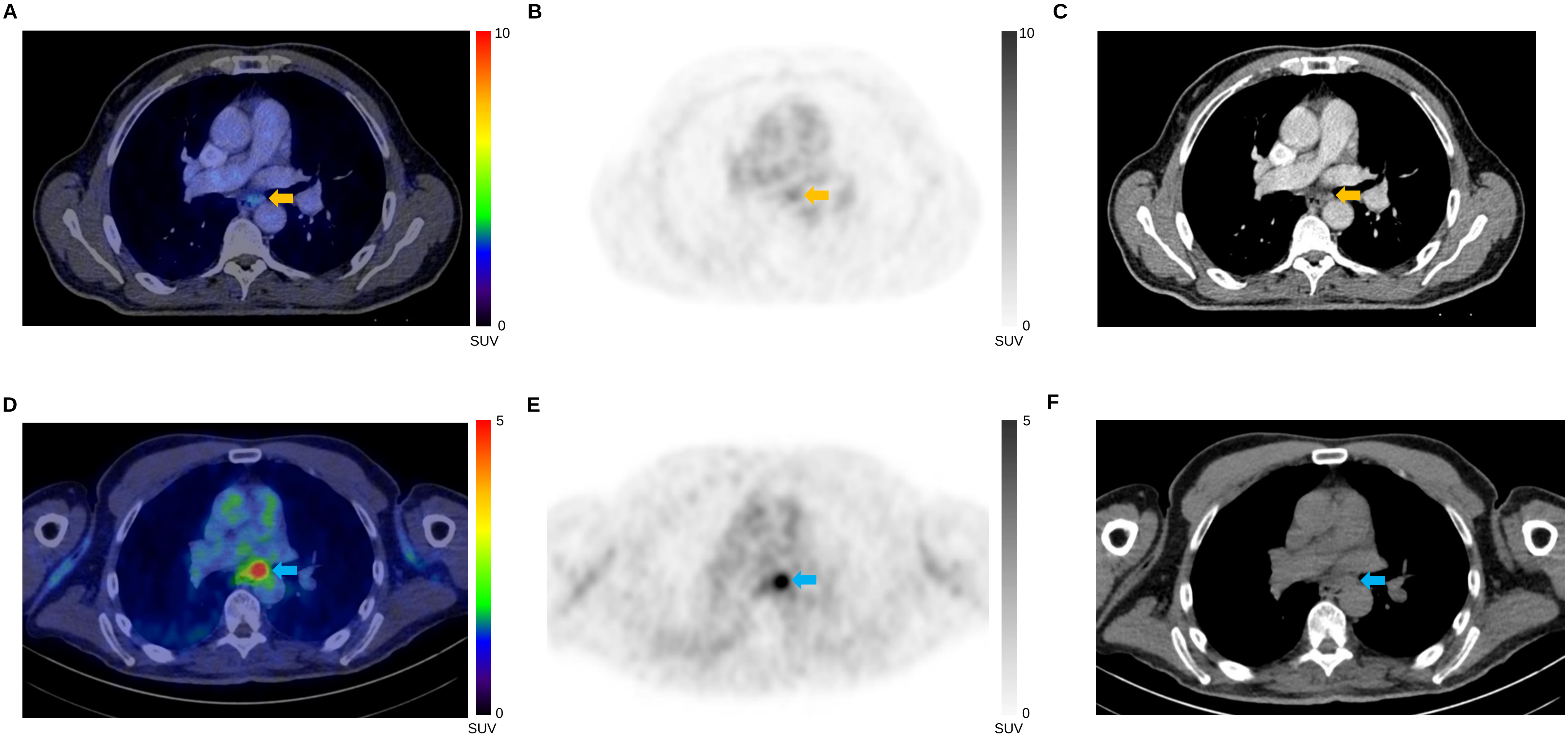

Preoperative [^{18}F]PSMA-1007 PET/CT (A-C) and follow-up [^{68}Ga]Ga-PSMA-I&T PET/CT 33 months later (D-F). Low PSMA expression of an infracarinary lymph node on the preoperative scan (visual score: 1, SUVmax: 4; yellow arrows) with increasing PSMA positivity on the follow-up PET/CT (visual score: 2, SUVmax: 7.2; blue arrows).

Pat. Nr. 8
Lesion 1 (IIR)

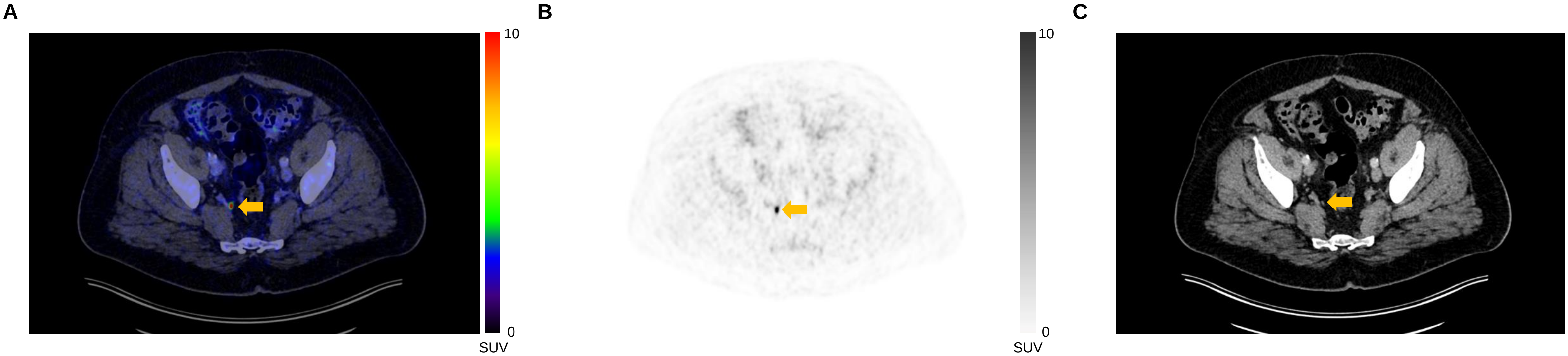

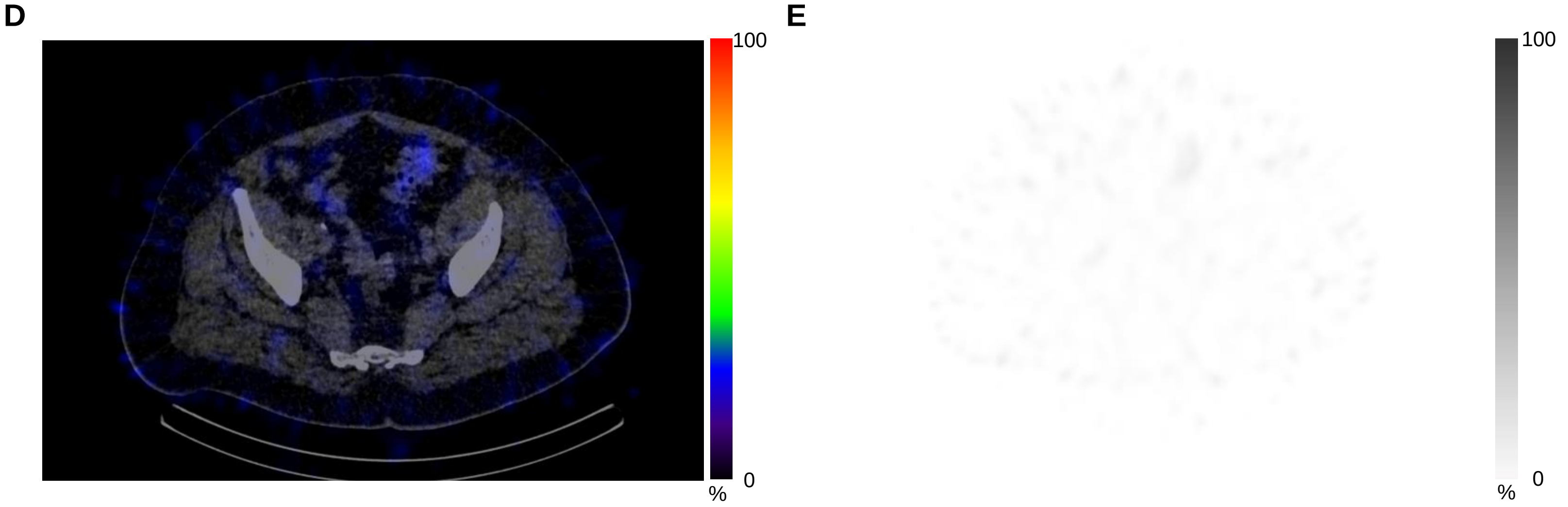

Axial [^{18}F]PSMA-1007 PET/CT (A-C) and [^{99m}Tc]Tc-PSMA-I&S (D,E). High PSMA expression of a lymph node adjacent to the right internal iliac artery (visual score: 3, SUVmax: 17.3; yellow arrows) without perceivable uptake on SPECT/CT.
Lesion status after retrospective analysis: true positive

Pat. Nr. 8

EIL and Tr bladder (additional lesions, follow-up)

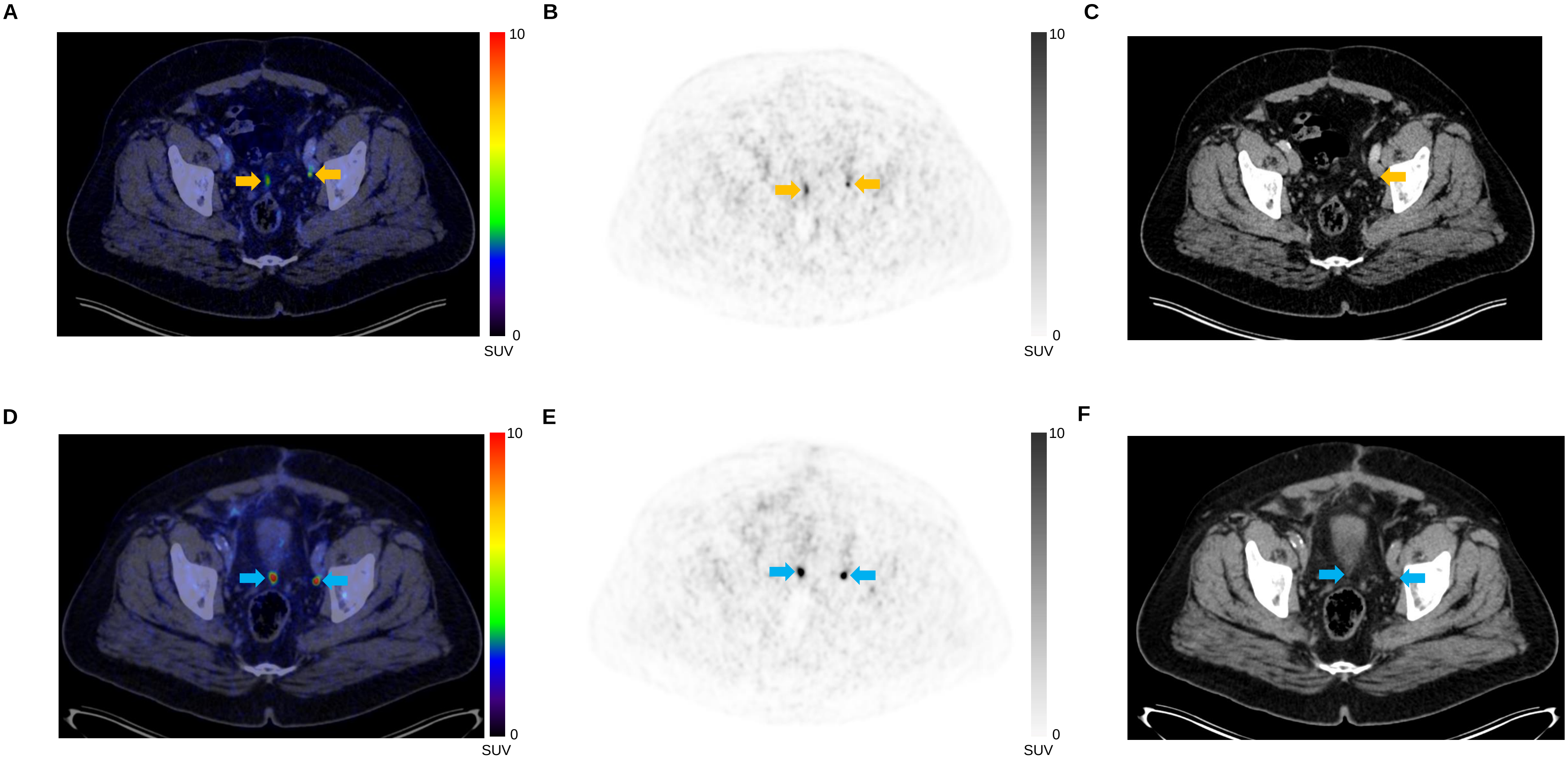

Preoperative [18F]PSMA-1007 PET/CT (A-C) with intermediate PSMA expression of a lymph node adjacent to the left external iliac artery (visual score 2, SUVmax: 8.5; yellow arrows) and low PSMA expression of a local recurrence which was morphologically indistinguishable from the urinary bladder wall (visual score 1, SUVmax: 7.8; yellow arrows). High PSMA expression of the same lesions on a follow-up [18F]PSMA-1007 PET/CT 5 months later (D-F; EIL: visual score 3, SUVmax: 26; Tr bladder: visual score 3, SUVmax 17.2; blue arrows). Lesion status after retrospective analysis: additional true positive

Pat. Nr. 8

Tr ductus deferens (additional lesion, follow-up)

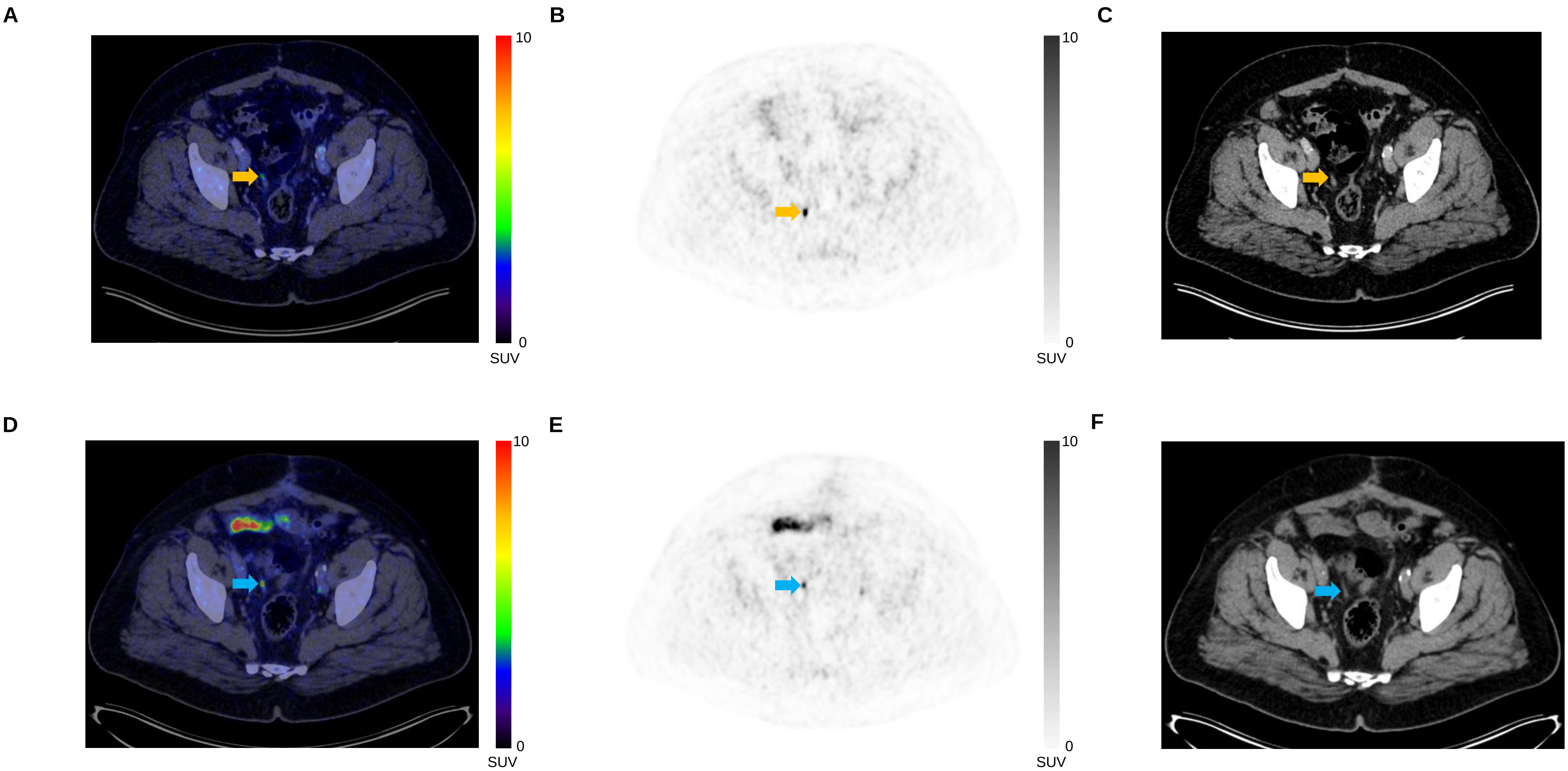

Preoperative [^{18}F]PSMA-1007 PET/CT (A-C) with low PSMA expression of a focal lesion of the ductus deferens (visual score 1; SUVmax: 4.9; yellow arrows). Intermediate PSMA expression of the same lesion on a follow-up [^{18}F]PSMA-1007 PET/CT 5 months later (D-F; visual score 2, SUVmax: 11.1; blue arrows) . Lesion status after retrospective analysis: additional true positive

Pat. Nr. 9
Lesion 1 (Tr)

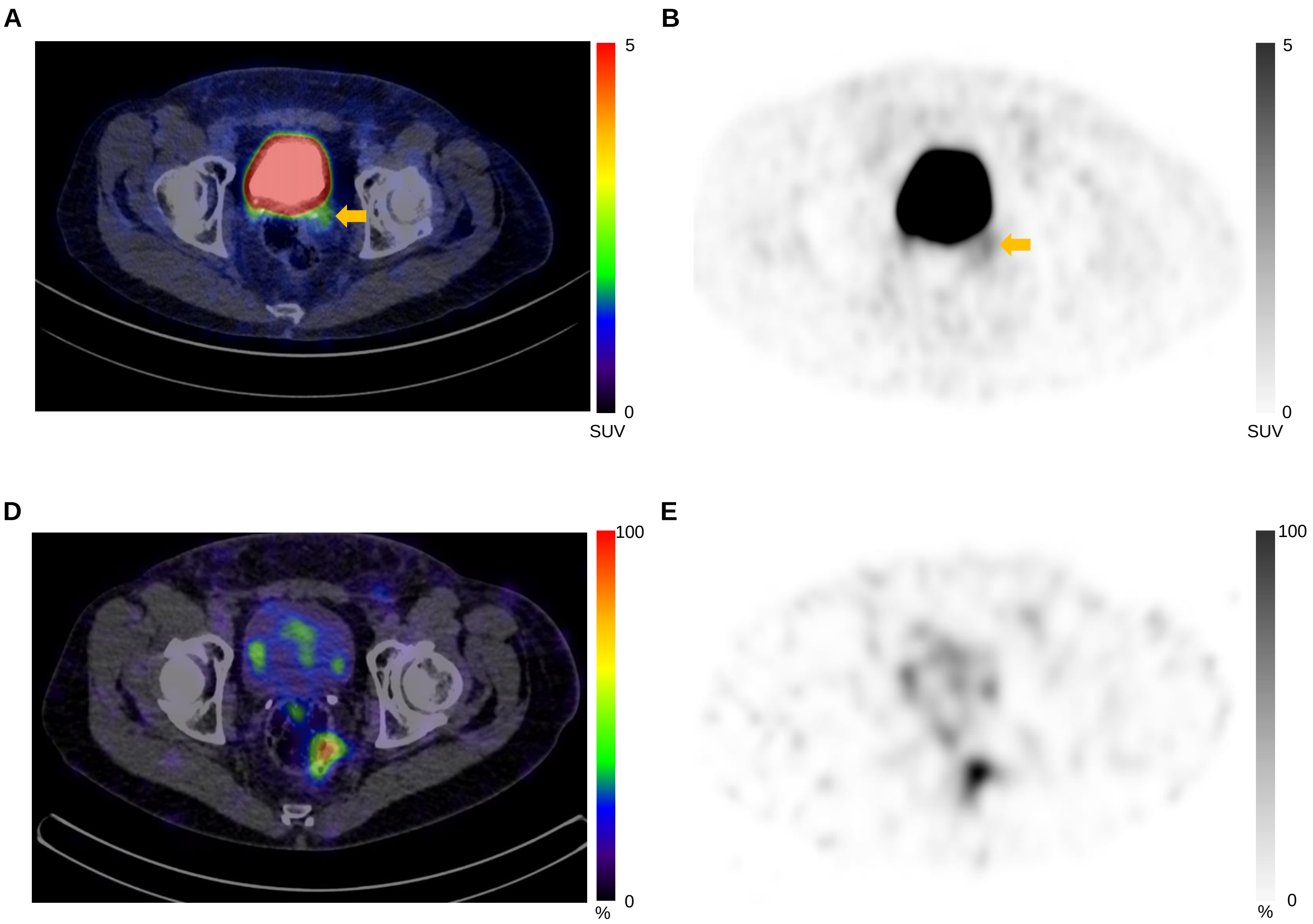

Axial [^{68}Ga]Ga-PSMA-I&T PET/CT (A-C) and [^{99m}Tc]Tc-PSMA-I&S SPECT/CT (D,E). Low PSMA expression of a focal lesion in the prostatic fossa on the left (visual score: 1, SUVmax: 1.9; yellow arrows) without perceiviable uptake on SPECT/CT.
Lesion status after retrospective analysis: false positive

Pat. Nr. 9

M1b (additional lesion, follow-up)

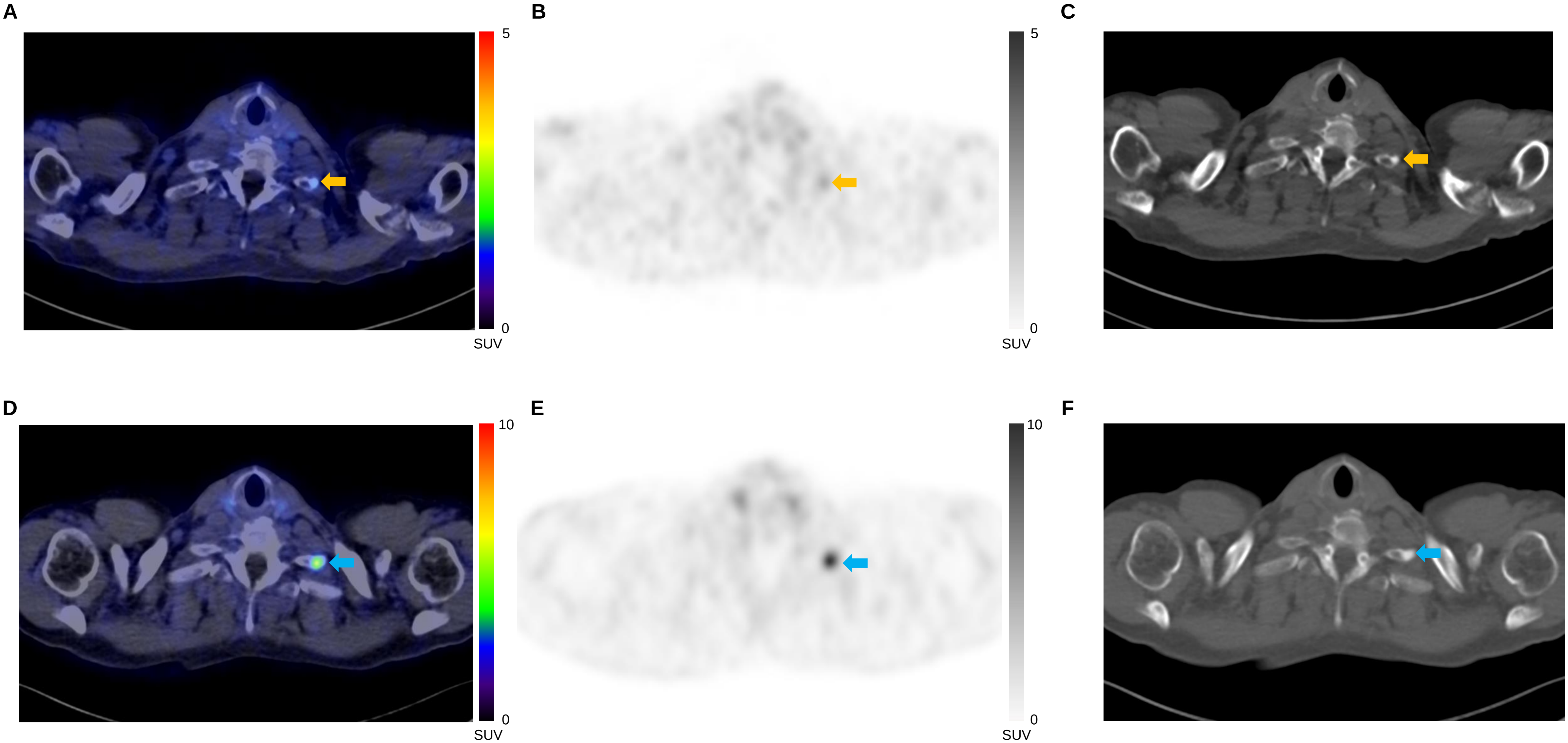

Preoperative [^{68}Ga]Ga-PSMA-I&T PET/CT (A-C) and follow-up [^{18}F]PSMA-1007 PET/CT 6 months later (D-F). Low PSMA expression of a sclerotic lesion of the first rib on the left on the preoperative scan (visual score: 1, SUVmax: 1.7; yellow arrows) with persistent uptake on the follow-up PET/CT (visual score: 2, SUVmax: 7.5; blue arrows). Lesion status after retrospective analysis: additional true positive

Pat. Nr. 10
Lesion 1 (EIR_1)

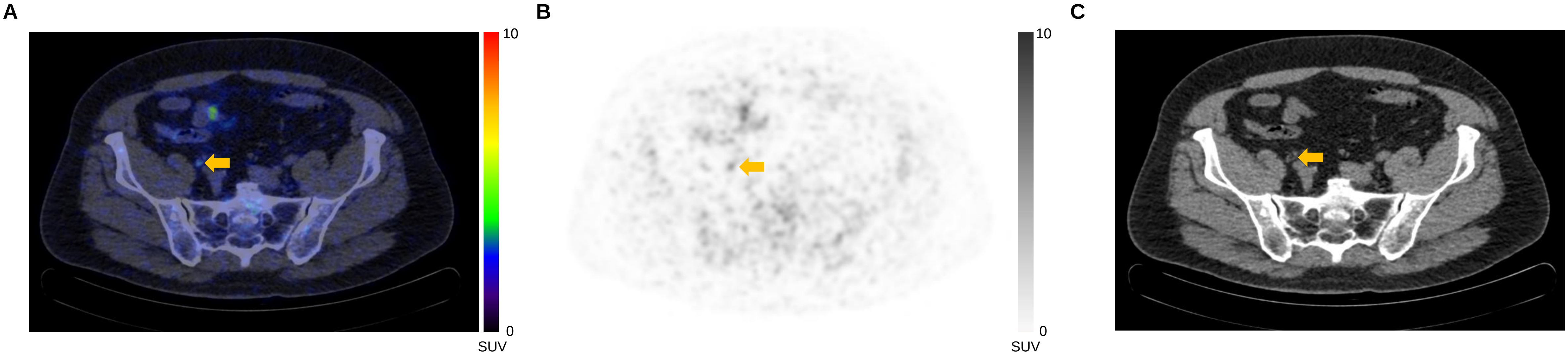

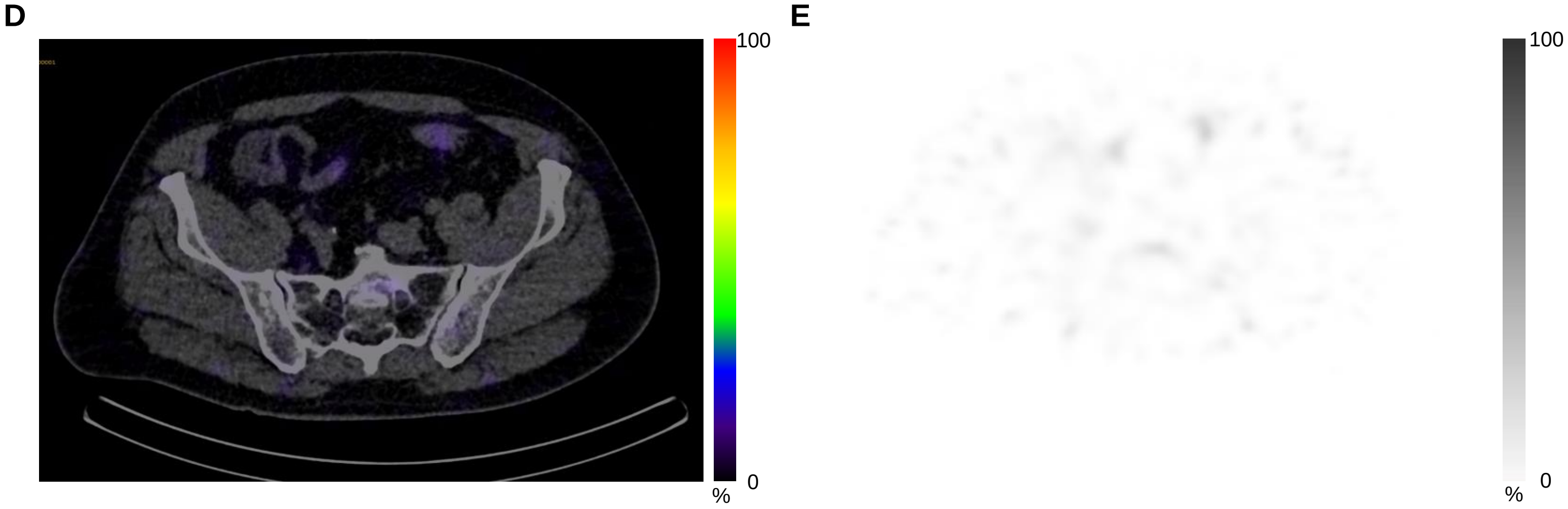

Axial [^{18}F]PSMA-1007 PET/CT (A-C) and [^{99m}Tc]Tc-PSMA-I&S SPECT/CT (D,E). Low PSMA expression of a lymph node adjacent to the right proximal external iliac artery (visual score: 1, SUVmax: 4; yellow arrows) without perceivable uptake on SPECT/CT.
Lesion status after retrospective analysis: unclear

Pat. Nr. 10
Lesion 2 (EIR_2)

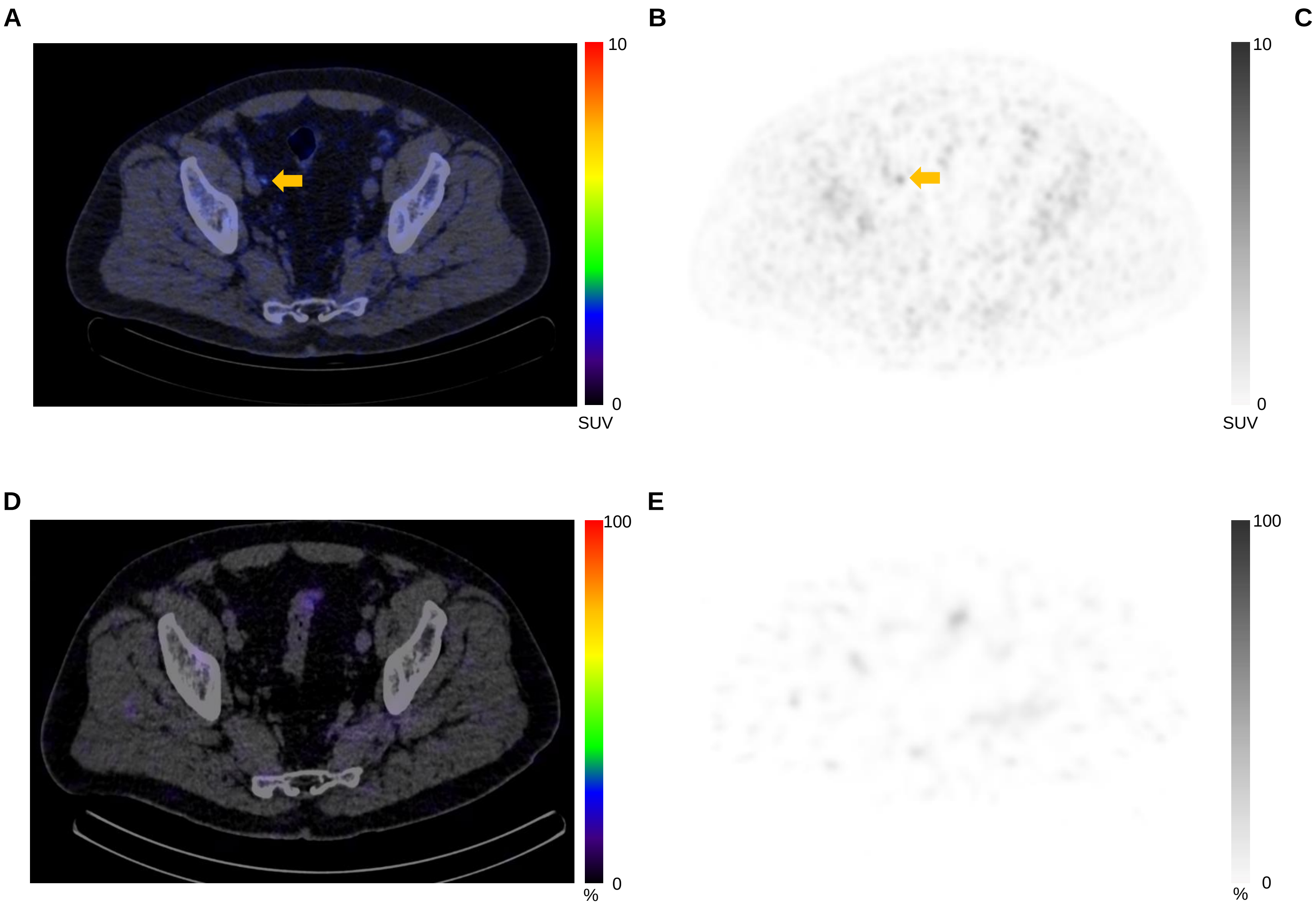

Axial [^{18}F]PSMA-1007 PET/CT (A-C) and [^{99m}Tc]Tc-PSMA-I&S SPECT/CT (D,E). Low PSMA expression of a lymph node adjacent to the right distal external iliac artery (visual score: 1, SUVmax: 3.2; yellow arrows) without perceivable uptake on SPECT/CT.
Lesion status after retrospective analysis: unclear

Pat. Nr. 10
Lesion 3 (CIL)

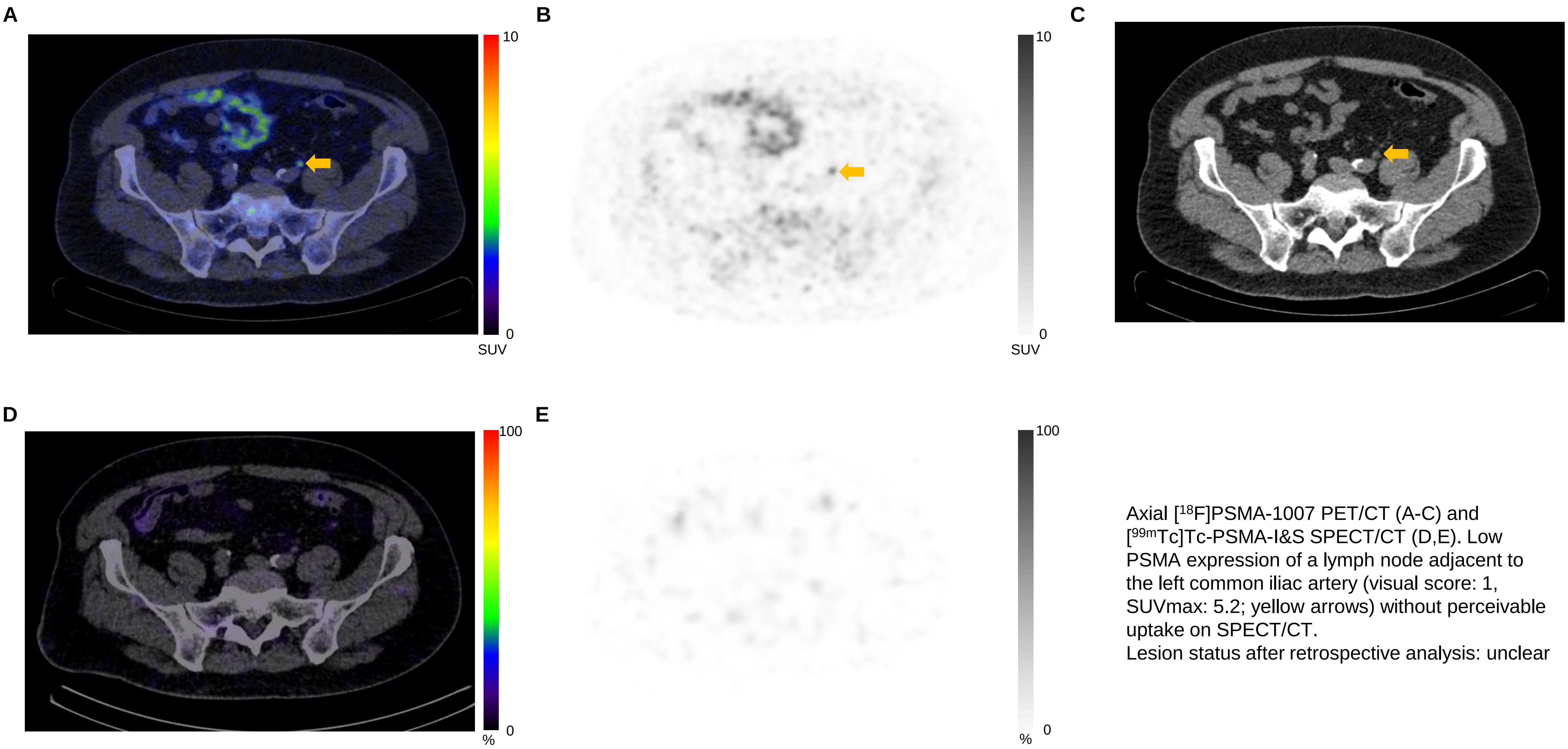

Axial [^{18}F]PSMA-1007 PET/CT (A-C) and [^{99m}Tc]Tc-PSMA-I&S SPECT/CT (D,E). Low PSMA expression of a lymph node adjacent to the left common iliac artery (visual score: 1, SUVmax: 5.2; yellow arrows) without perceivable uptake on SPECT/CT.
Lesion status after retrospective analysis: unclear

Pat. Nr. 11
Lesion 1 (IIL)

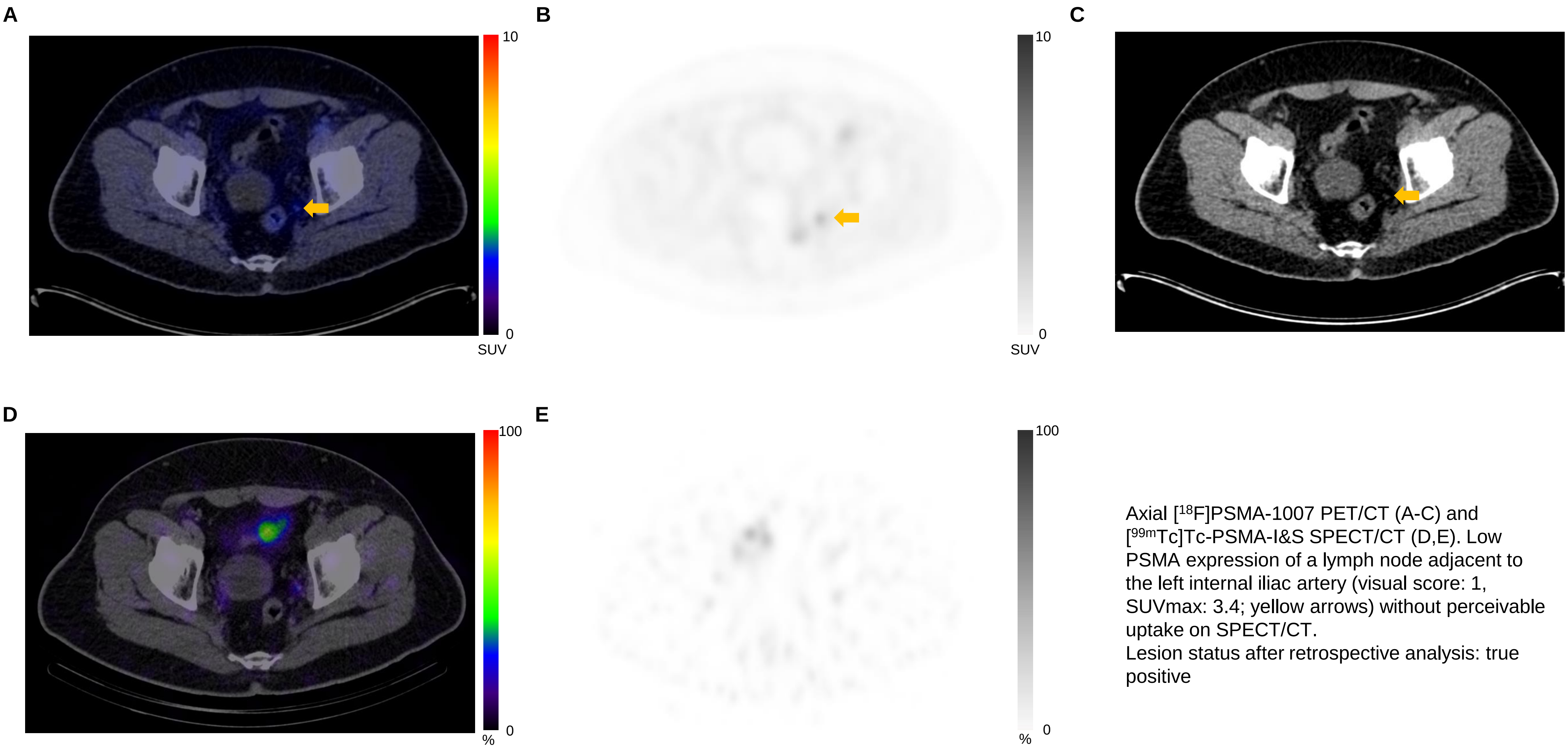

Axial [^{18}F]PSMA-1007 PET/CT (A-C) and [^{99m}Tc]Tc-PSMA-I&S SPECT/CT (D,E). Low PSMA expression of a lymph node adjacent to the left internal iliac artery (visual score: 1, SUVmax: 3.4; yellow arrows) without perceivable uptake on SPECT/CT.
Lesion status after retrospective analysis: true positive

Pat. Nr. 11
Lesion 1 (IIL, follow-up)

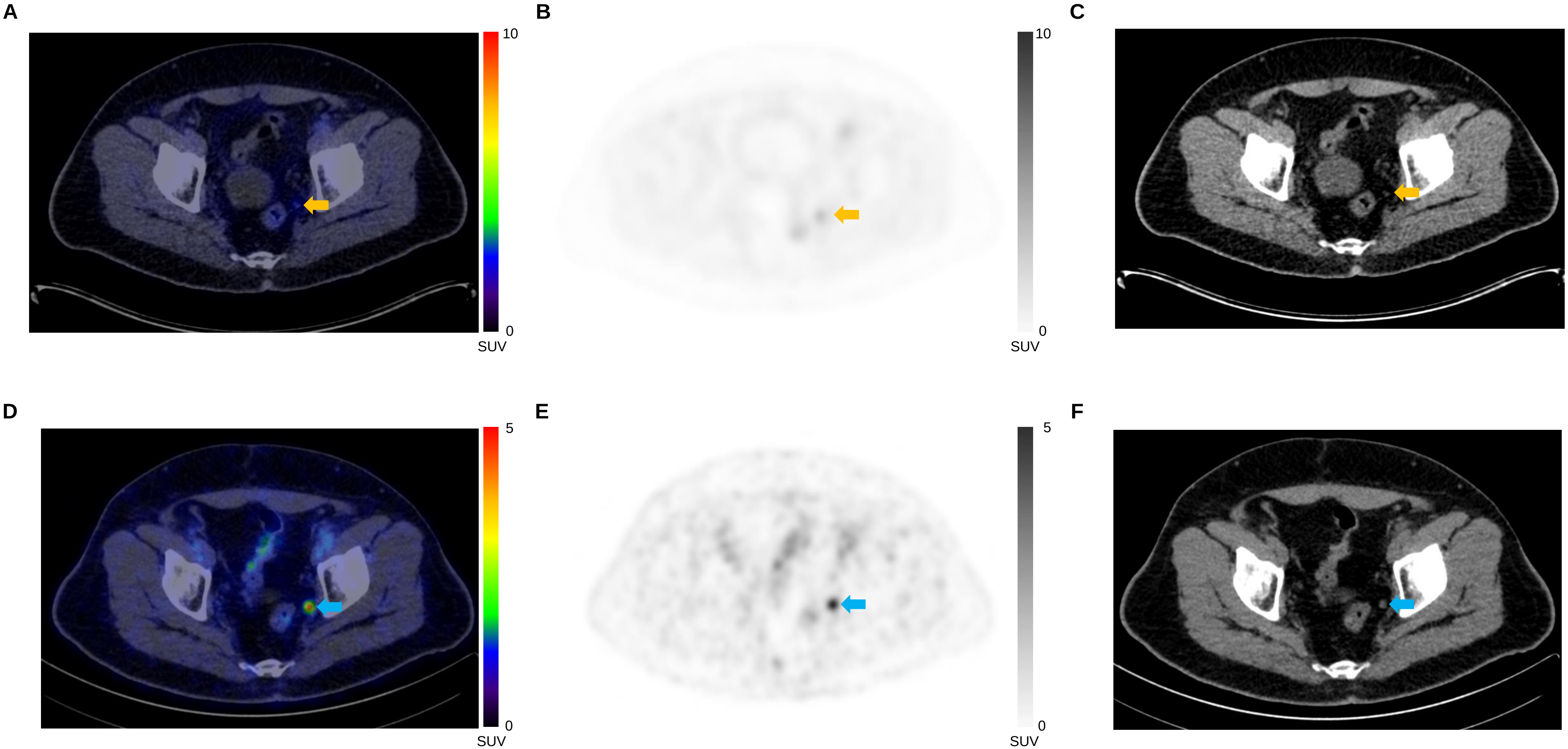

Preoperative [^{18}F]PSMA-1007 PET/CT (A-C) and follow-up [^{68}Ga]Ga-PSMA-I&T PET/CT 31 months later (D-F). Low PSMA expression of a lymph node adjacent to the left internal iliac artery on the preoperative scan (visual score 1, SUVmax: 3.4; yellow arrows) with persistent uptake on the follow-up PET/CT (visual score 2, SUVmax: 4.9; blue arrows). Lesion status after retrospective analysis: true positive

Pat. Nr. 12
Lesion 1 (IIL)

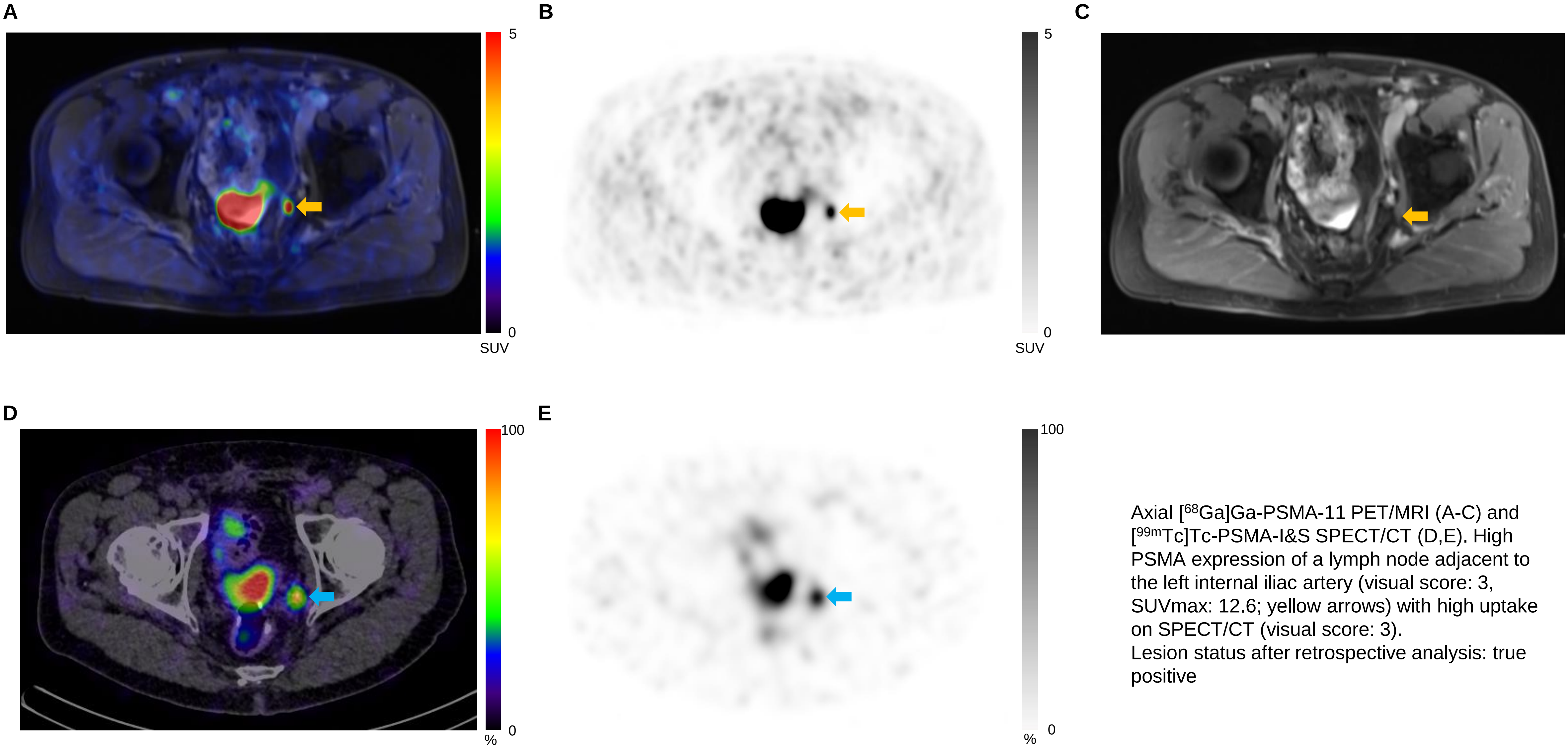

Axial [^{68}Ga]Ga-PSMA-11 PET/MRI (A-C) and [^{99m}Tc]Tc-PSMA-I&S SPECT/CT (D,E). High PSMA expression of a lymph node adjacent to the left internal iliac artery (visual score: 3, SUVmax: 12.6; yellow arrows) with high uptake on SPECT/CT (visual score: 3).
Lesion status after retrospective analysis: true positive

Pat. Nr. 12
Lesion 1 (IIL, follow-up)

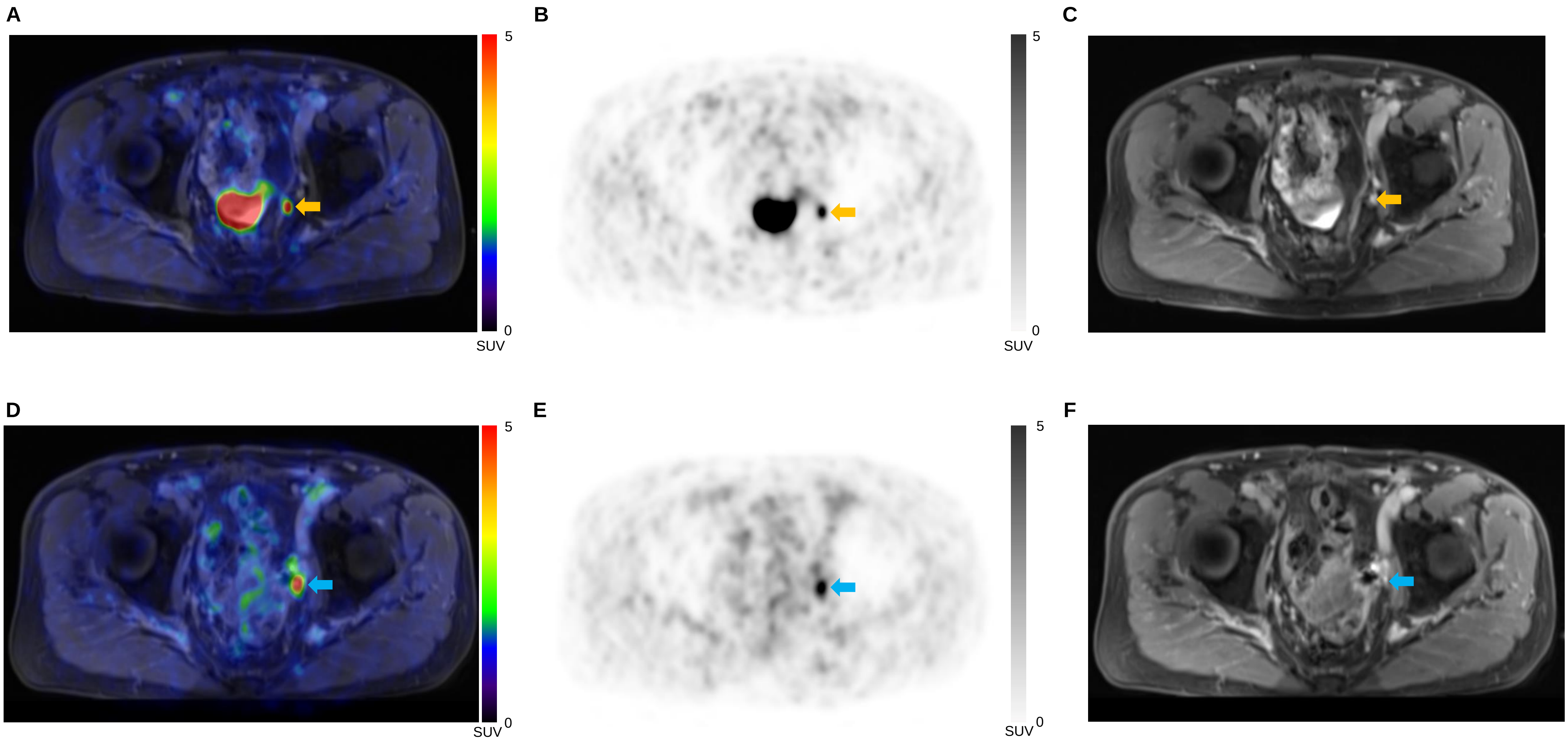

Preoperative [68Ga]Ga-PSMA-11 PET/MRI (A-C) and follow-up [68Ga]Ga-PSMA-11 PET/MRI 13 months later (D-F). High PSMA expression of a lymph node next to the left internal iliac artery on the preoperative scan (visual score: 3, SUVmax: 12.6; yellow arrows) with persistent uptake on the follow-up PET/MRI (visual score: 2, SUVmax: 7.7; blue arrows). Lesion status after retrospective analysis: true positive

Pat. Nr. 13

No lesion – false positive result due to high urine activity next to a lymph node

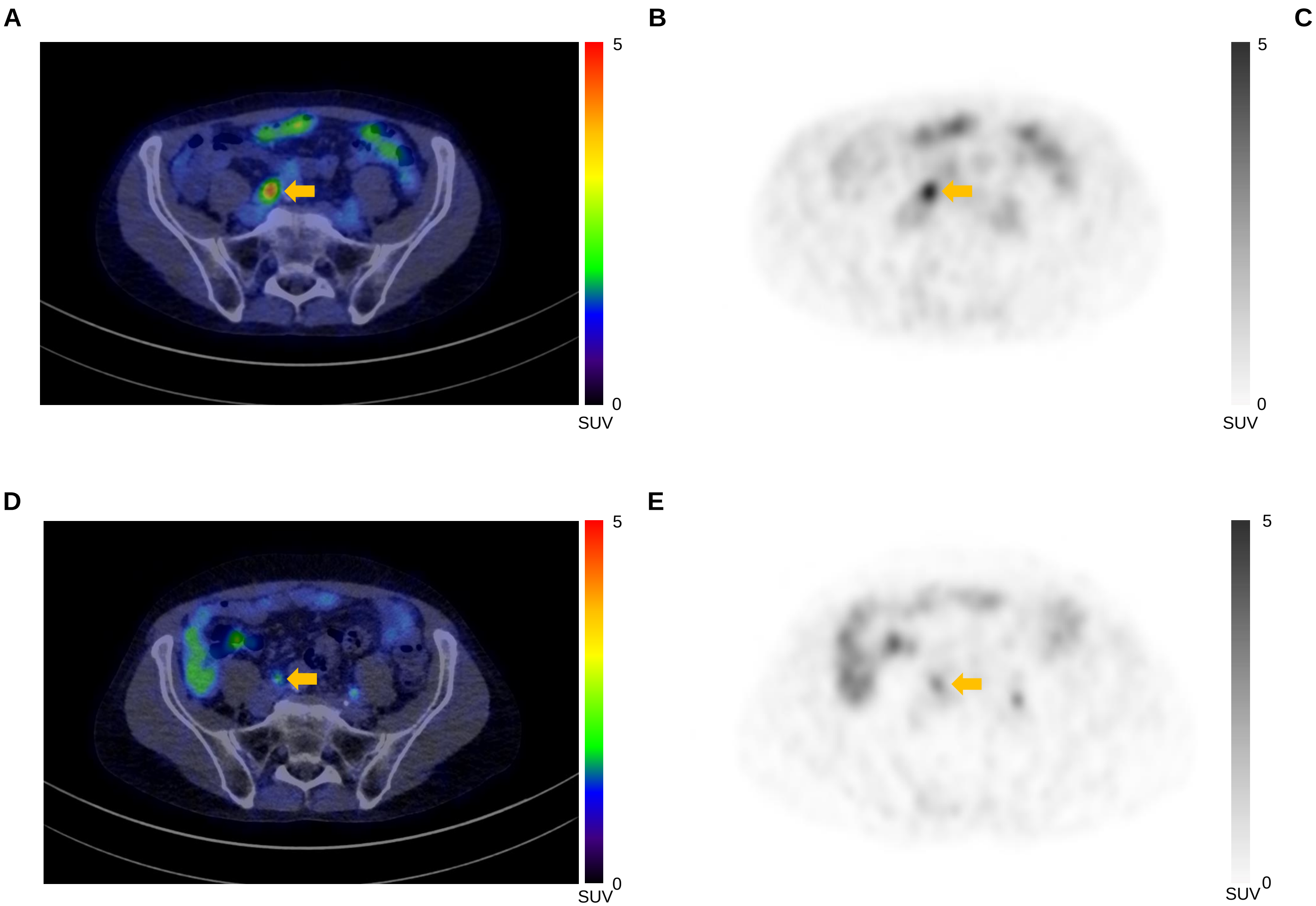

Axial [^{68}Ga]Ga-PSMA-I&T PET/CT with an early scan (60 min. uptake time, A-C) and additional late scan of the pelvis (180. min. uptake time, D – E). High urine activity in the right ureter due to renal excretion of [^{68}Ga]Ga-PSMA-I&T (yellow arrows). A prominent lymph node adjacent to the right ureter (blue arrow) was falsly interpreted as a lymph node metastasis.

Lesion status after retrospective analysis: false positive

Pat. Nr. 14
Lesion 1 (CIL)

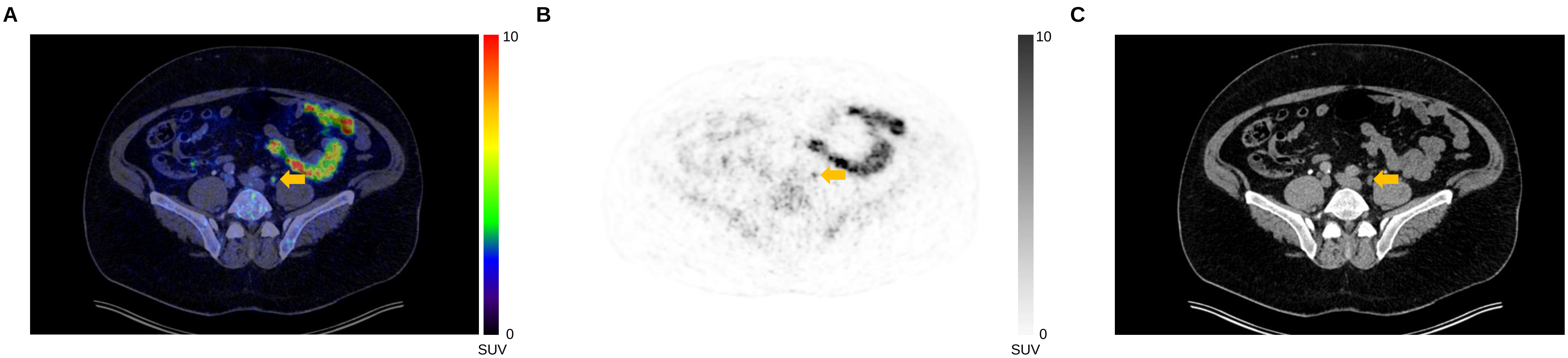

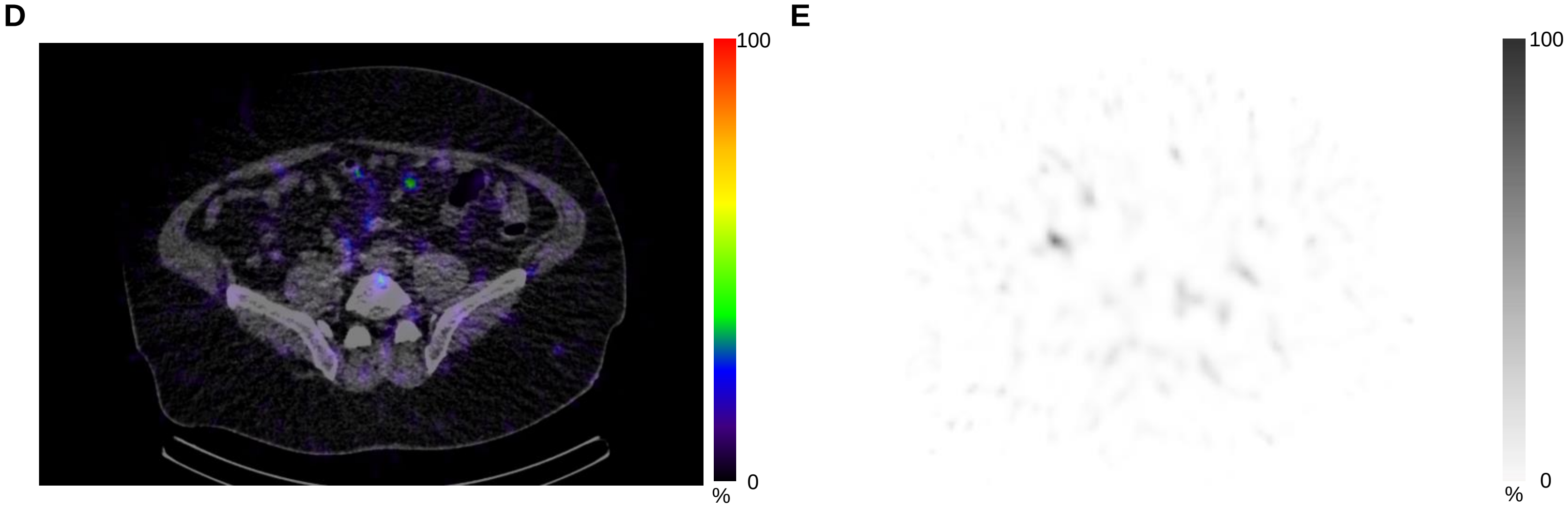

Axial [^{18}F]PSMA-1007 PET/CT (A-C) and [^{99m}Tc]Tc-PSMA-I&S SPECT/CT (D,E). Low PSMA expression of a lymph node next to the left common iliac artery (visual score: 1, SUVmax: 6.1; yellow arrows) without perceivable uptake on SPECT/CT.
Lesion status after retrospective analysis: unclear

Pat. Nr. 15
Lesion 1 (EIL)

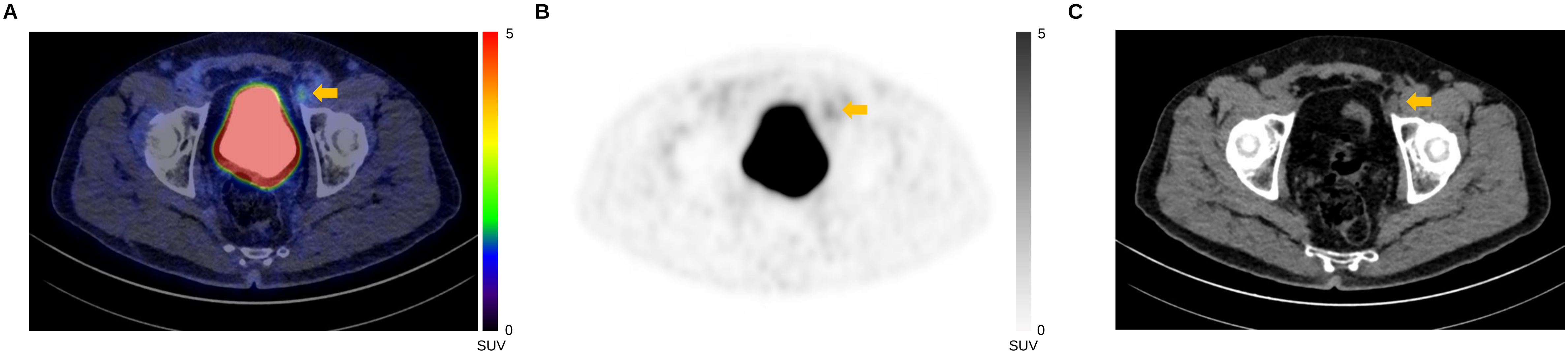

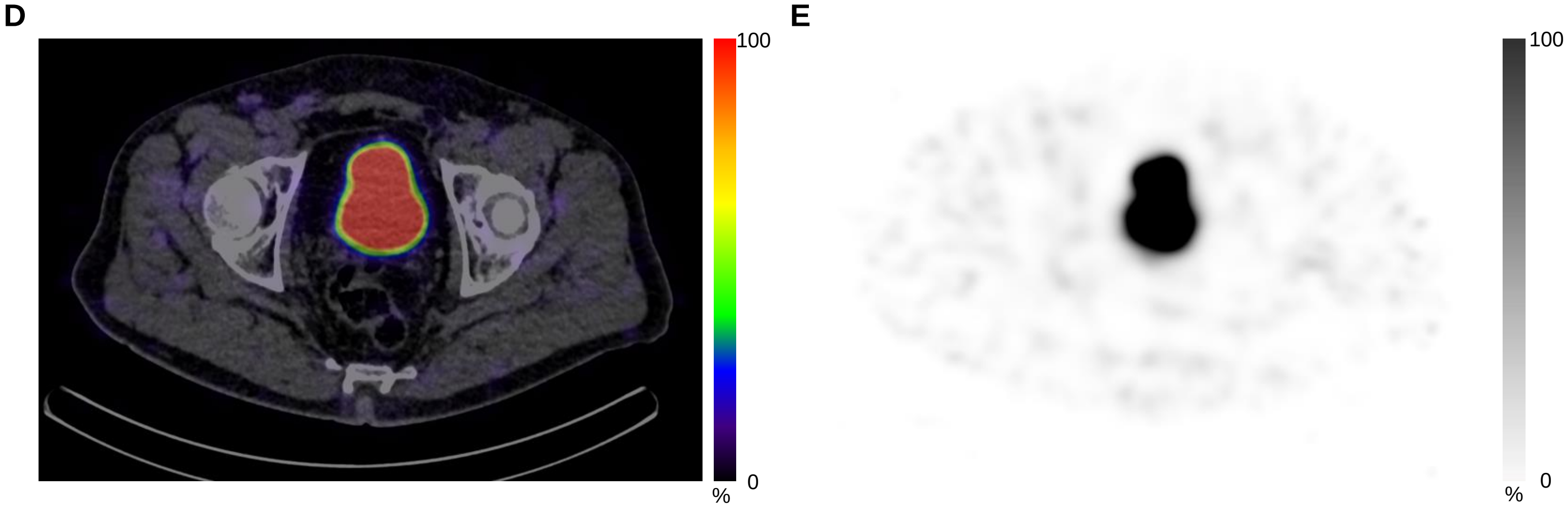

Axial [^{68}Ga]Ga-PSMA-I&T PET/CT (A-C) and [^{99m}Tc]Tc-PSMA-I&S SPECT/CT (D,E). Low PSMA expression of a lymph node adjacent to the left external iliac vessels (visual score: 1, SUVmax: 2.4; yellow arrows) without uptake on SPECT/CT.
Lesion status after retrospective analysis: false positive

Pat. Nr. 15

Tr (additional lesion, follow-up)

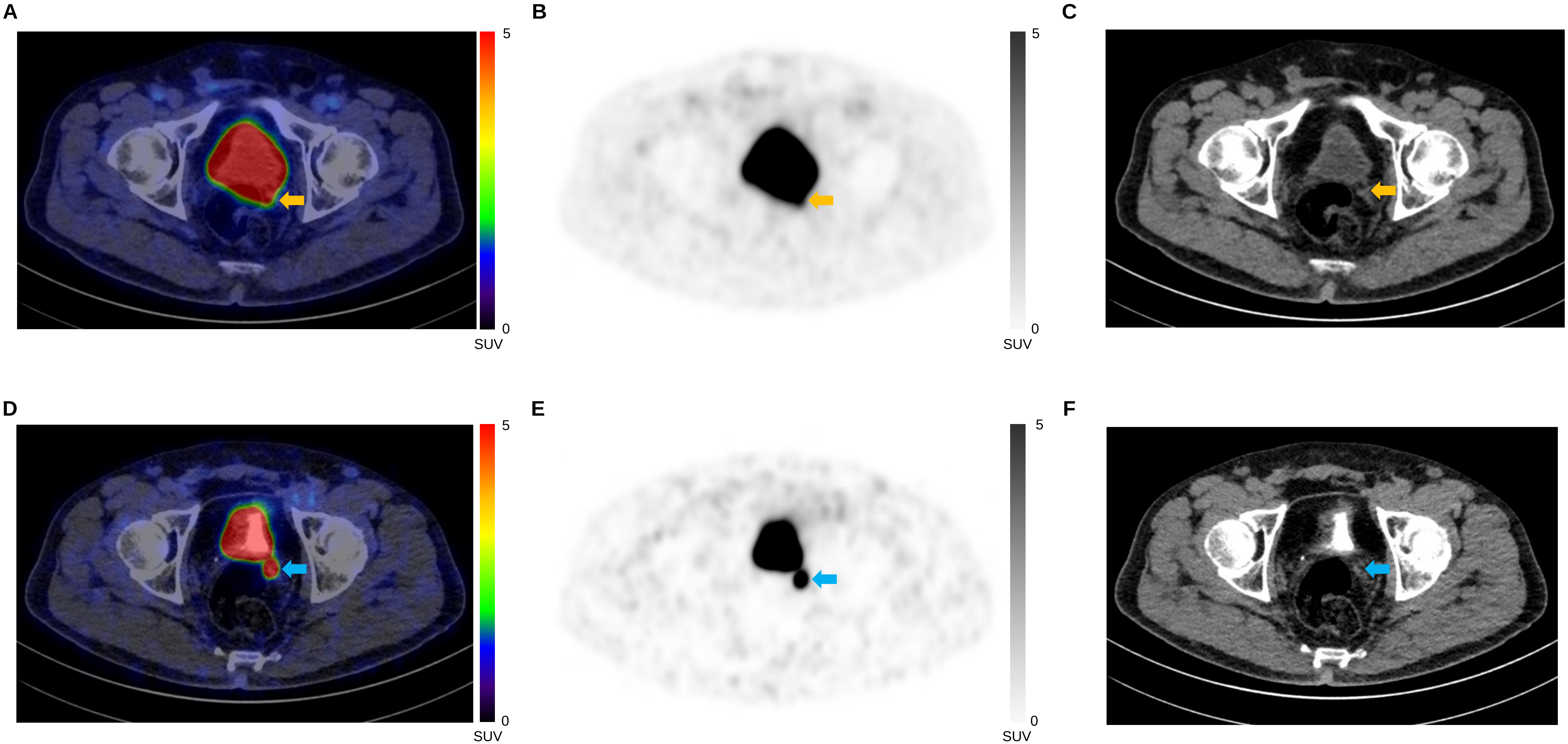

Preoperative [^{68}Ga]Ga-PSMA-I&T PET/CT (A-C) and follow-up [^{68}Ga]Ga-PSMA-I&T PET/CT 12 months later (D-F). PSMA expression of a focal lesion of the prostatic fossa masked by high urine activity on the preoperative scan (SUVmax: not reliably measurable; yellow arrows) with distinct uptake on the follow-up PET/CT (visual score 3, SUVmax: 14.4; blue arrows). Lesion status after retrospective analysis: additional true positive

Pat. Nr. 16
Lesion 1 (Tr)

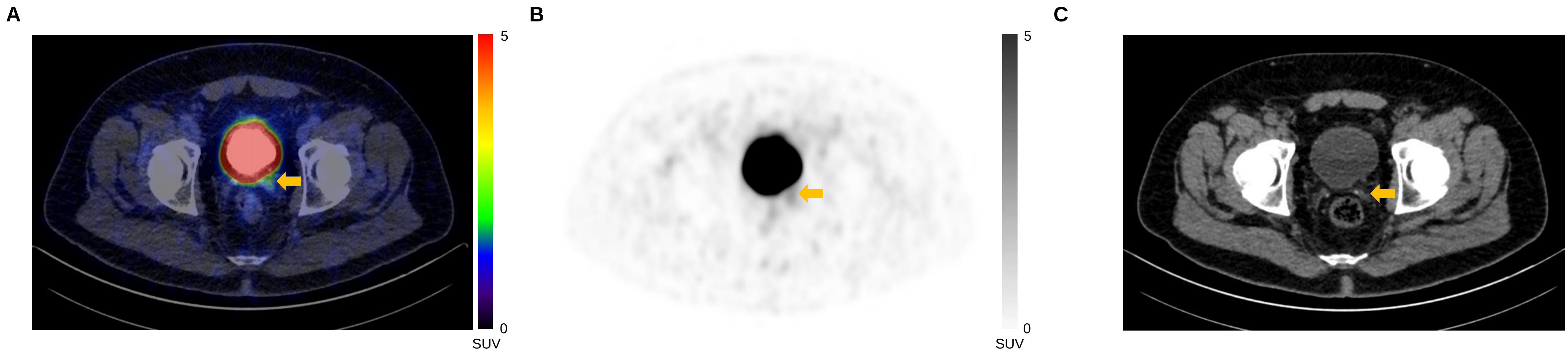

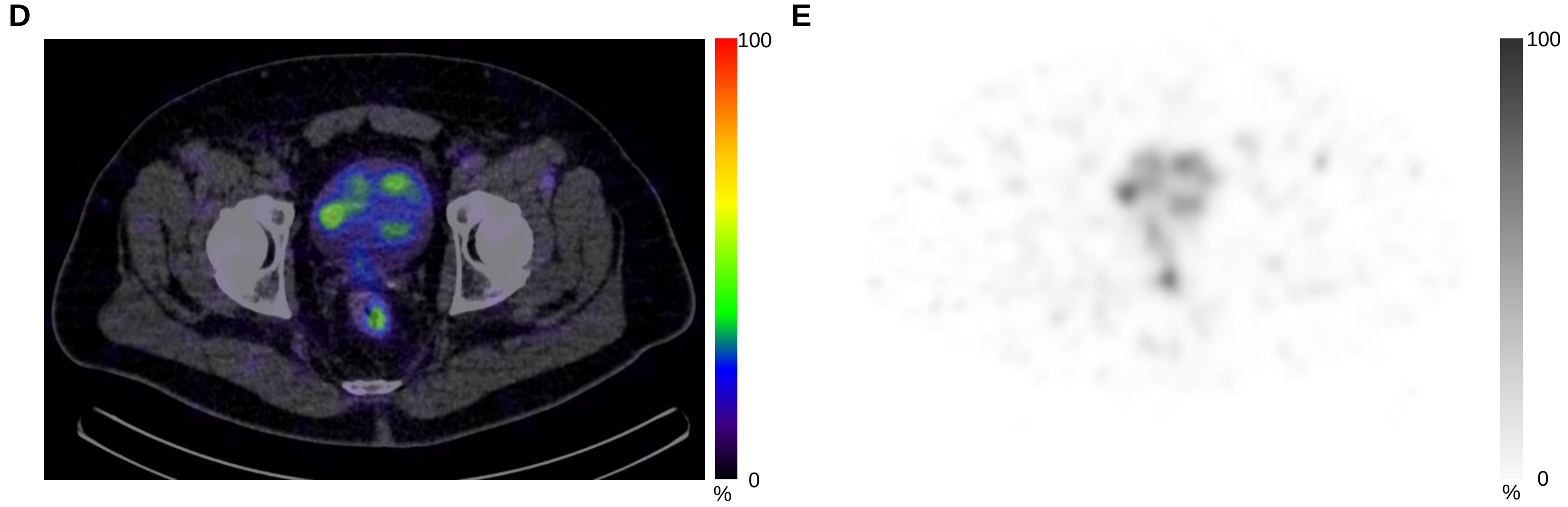

Axial [^{68}Ga]Ga-PSMA-I&T PET/CT (A-C) and [^{99m}Tc]Tc-PSMA-I&S SPECT/CT (D,E). Low PSMA expression of a focal lesion in the prostatic fossa on the left (visual score: 1, SUVmax: 2.2; arrows) without perceivable uptake on SPECT/CT.
Lesion status after retrospective analysis: false positive

Pat. Nr. 16

Tr follow-up

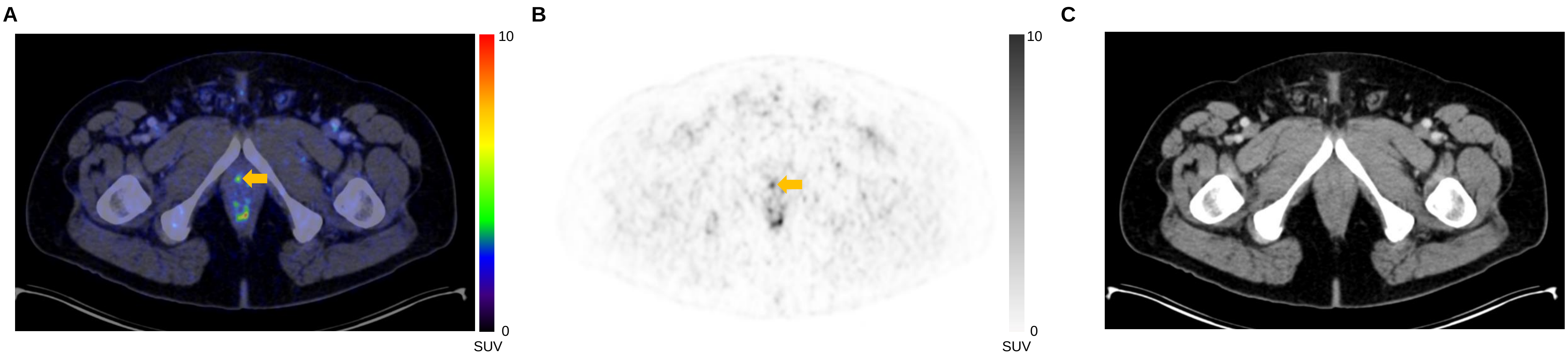

Axial follow-up [^{18}F]PSMA-1007 (A-C). Low PSMA expression of a lesion in the prostatic fossa adjacent to the anastomosis (visual score 1, SUVmax: 7.2; yellow arrows) without a correlate on CT.

Pat. Nr. 17
Lesion 1 (Tr)

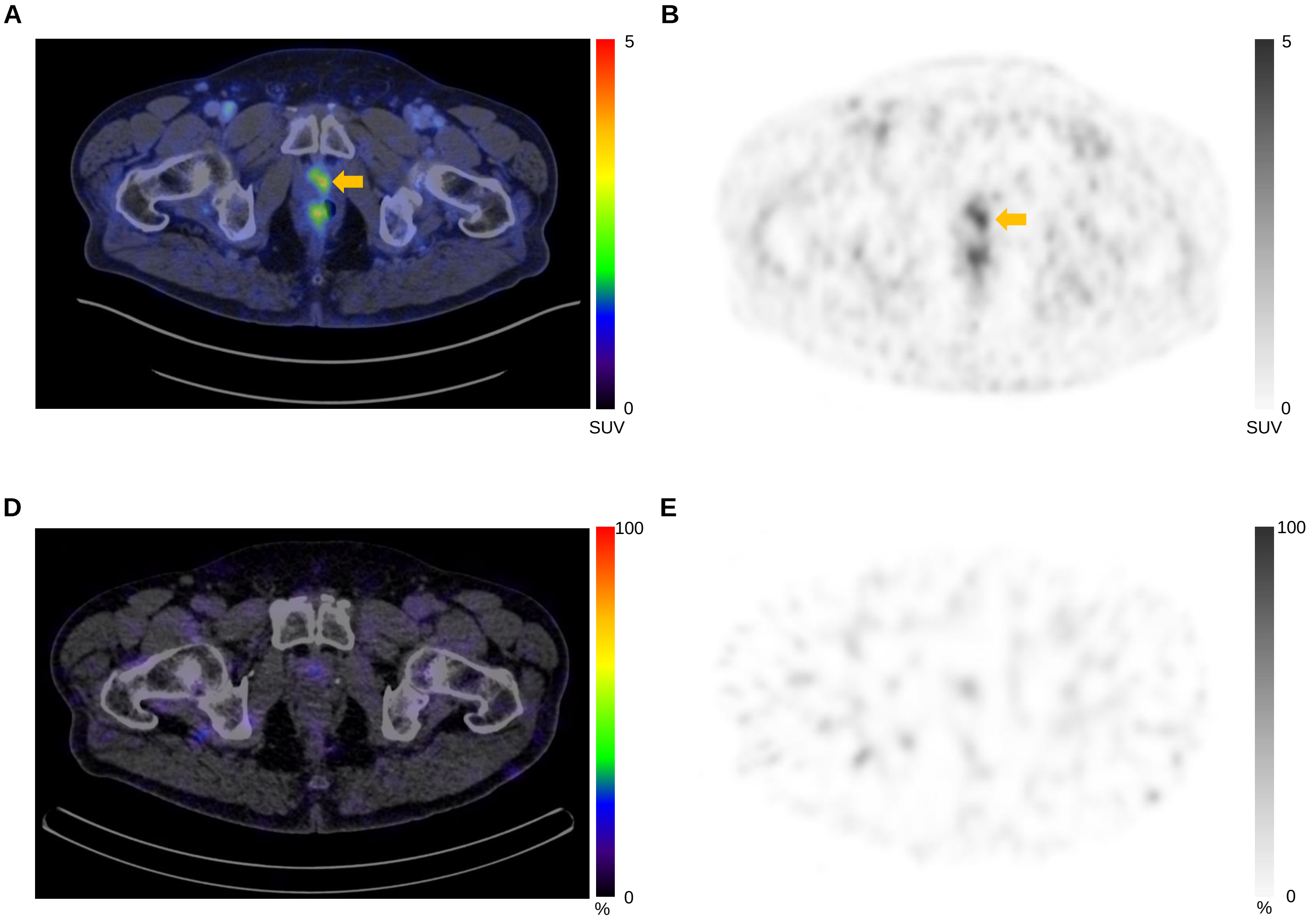

Axial [^{68}Ga]Ga-PSMA-11 PET/CT (A-C) and [^{99m}Tc]Tc-PSMA-I&S SPECT/CT (D,E). Low PSMA expression of a focal lesion in the prostatic fossa adjacent to the anastomisis (visual score: 1; SUVmax: 3.9; yellow arrows) without perceivable uptake on SPECT/CT. Lesion status after retrospective analysis: true positive

Pat. Nr. 17
Lesion 2 (CIL)

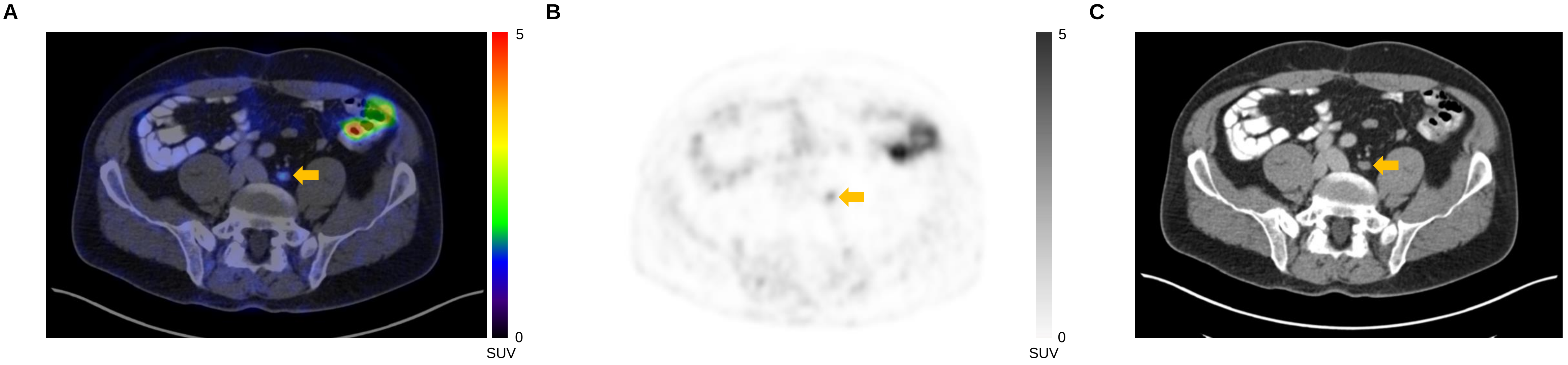

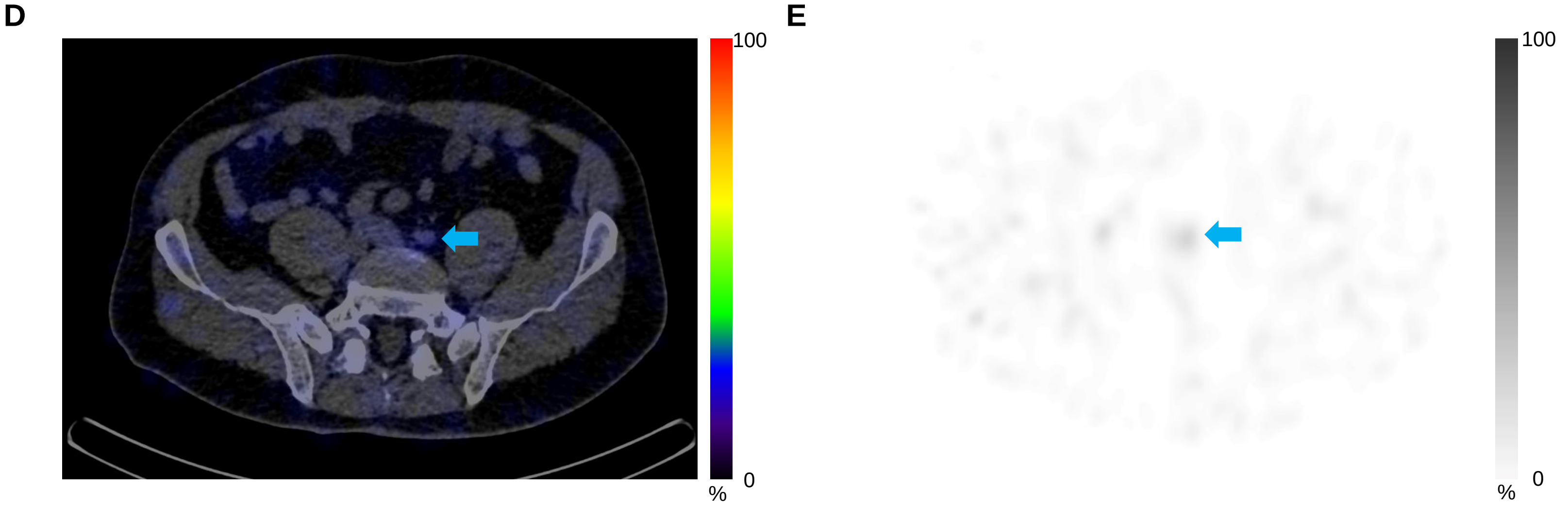

Axial [^{68}Ga]Ga-PSMA-11 PET/CT (A-C) and [^{99m}Tc]Tc-PSMA-I&S SPECT/CT (D,E). Low PSMA expression of a lymph node next to the left common iliac artery (visual score: 1,SUVmax: 2.6; yellow arrows) with low uptake on SPECT/CT (visual score 1; blue arrows). Lesion status after retrospective analysis: false positive

Pat. Nr. 17
Leison 1 (Tr, follow-up)

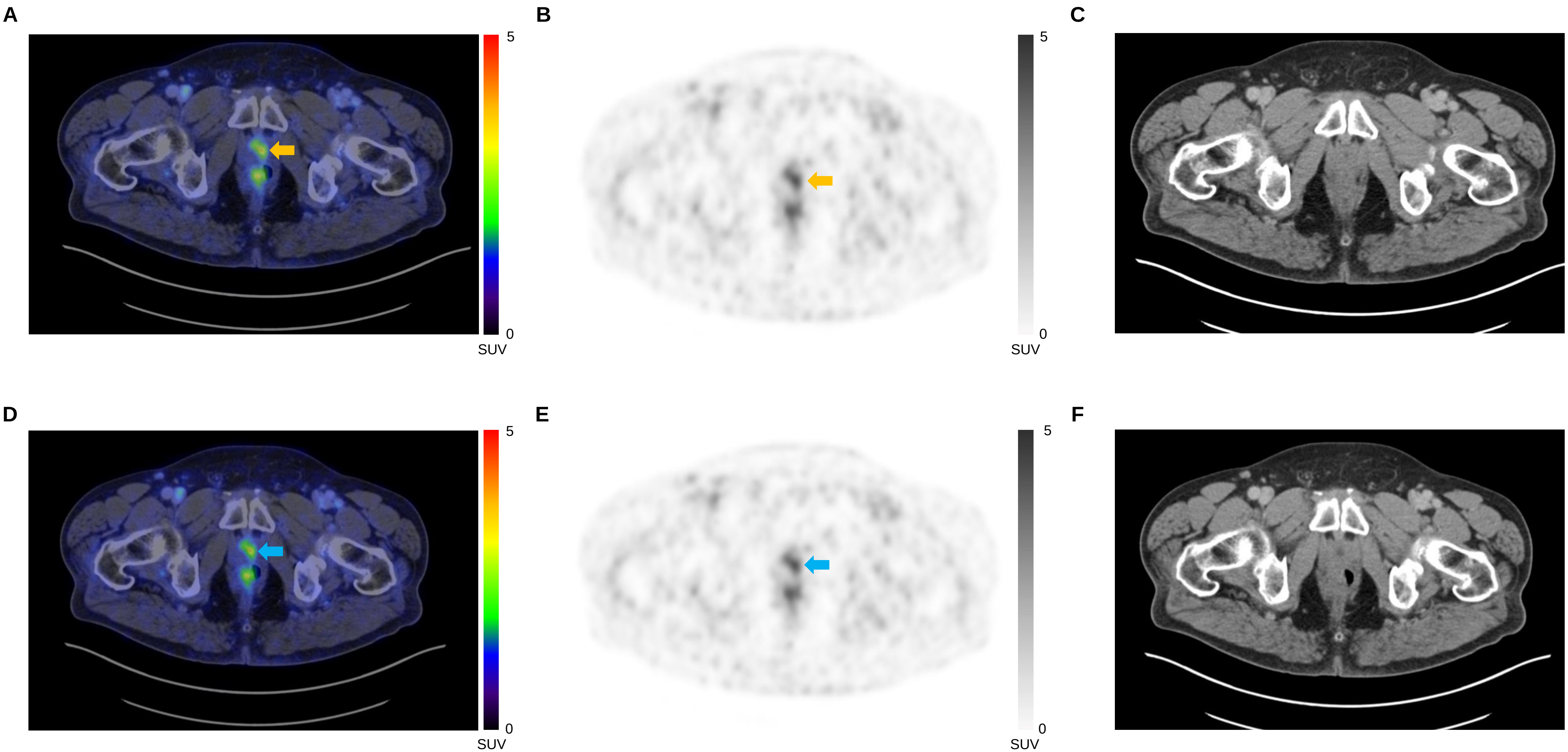

Preoperative [^{68}Ga]Ga-PSMA-11 PET/CT (A-C) and follow-up [^{68}Ga]Ga-PSMA-11 PET/CT 7 months later (D-F). Low PSMA expression of a focal lesion in the prostatic fossa next to the anastomisis (visual score: 1; SUVmax: 3.9; yellow arrows) with persistent uptake on the follow-up PET/CT (visual score: 1, SUVmax: 4.3; blue arrows). Lesion status after retrospective analysis: true positive
