# Supplementary material for: Prostate-specific membrane antigen radioguided surgery with negative histopathology: an in-depth analysis
Source: Eur J Nucl Med Mol Imaging. 2023 Sep 26;51(2):548–57. doi: 10.1007/s00259-023-06442-7 (PMC10774205; doi:10.1007/s00259-023-06442-7)
Supplement: Supplementary file 2 — Supplementary file2 (DOCX 26 KB) [file 259_2023_6442_MOESM2_ESM.docx]

| **Supplementary Table 1** PET imaging features of index lesions and additional lesions identified on retrospective review. | | | | | | | | | | | | | |
| --- | --- | --- | --- | --- | --- | --- | --- | --- | --- | --- | --- | --- | --- |
| **Pat**. | Radioligand | Activity PET (MBq) | Lesion location | Short axis (mm) | Long axis (mm) | SUVmax | SUVmax to BG | Visual Score PET | Activity SPECT (MBq) | Visual Score SPECT | LN resection pelvis | LN resection retroperitoneum | Status after retrospective analysis |
| **1** | [^18^F]rhPSMA-7 | 313 | CIL | 7 | 8 | 3.1 | 4.7 | 1 | 690 | 0 | left | left | Unclear |
|  |  |  | EIL | 5 | 11 | 4.1 | 6.3 | 1 |  | 0 |  |  | Unclear |
| **2** | [^68^Ga]Ga-PSMA-I&T | 159 | RP_1 | 8 | 17 | 2.3 | 7.5 | 1 | 759 | 0 | right | both | FP |
|  |  |  | RP_2 | 8 | 17 | 1.6 | 5.3 | 1 |  | 0 |  |  | FP |
|  |  |  | *Tr* | *NA* | *NA* | *5* | *16.8* | *2* |  | *0* |  |  | *AD TP* |
| **3** | [^68^Ga]Ga-PSMA-11 | 128 | IIR | 9 | 18 | 11.9 | 29.8 | 3 | 752 | 3 | right | right | TP |
| **4** | [^18^F]PSMA-1007 | 396 | EIL | 9 | 19 | 3.9 | 9.2 | 1 | 713 | 0 | left | none | FP |
|  |  |  | Tr | NA | NA | 8.1 | 19.3 | 1 |  | 0 |  |  | FP |
| **5** | [^18^F]PSMA-1007 | 267 | EIL_1 | 8 | 18 | 4.3 | 8.4 | 1 | 630 | 0 | both | both | FP |
|  |  |  | EIL_2 | 10 | 22 | 5 | 9.7 | 1 |  | 0 |  |  | FP |
|  |  |  | *Tr* | *NA* | *NA* | *4.2* | *8.2* | *1* |  | *0* |  |  | *AD TP* |
| **6** | [^18^F]rhPSMA-7 | 388 | Tr | 8 | 10 | 6 | 11.1 | 1 | 658 | 3 | right | right | TP |
|  |  |  | EIR | 8 | 15 | 6 | 11.1 | 1 |  | 1 |  |  | FP |
| **7** | [^18^F]PSMA-1007 | 332 | CIL | 5 | 6 | 2.7 | 5.3 | 1 | 775 | 0 | left | left | TN |
| **8** | [^18^F]PSMA-1007 | 419 | IIR | 3 | 6 | 17.3 | 31 | 3 | 745 | 0 | right | right | TP |
|  |  |  | *EIL* | *3* | *5* | *8.5* | *15.3* | *2* |  | *0* |  |  | *AD TP* |
|  |  |  | *Tr ductus deferens* | *NA* | *NA* | *4.9* | *8.7* | *1* |  | *0* |  |  | *AD TP* |
|  |  |  | *Tr bladder* | *NA* | *NA* | *7.8* | *14* | *1* |  | *0* |  |  | *AD TP* |
| **9** | [^68^Ga]Ga-PSMA-I&T | 143 | Tr | NA | NA | 1.9 | 6.2 | 1 | 717 | 0 | both | none | FP |
|  |  |  | *M1b* | *NA* | *NA* | *1.7* | *5.5* | *1* |  | *0* |  |  | *AD TP* |
| **10** | [^18^F]PSMA-1007 | 211 | EIR_1 | 6 | 15 | 4 | 9.6 | 1 | 761 | 0 | both | both | Unclear |
|  |  |  | EIR_2 | 4 | 10 | 3.2 | 7.8 | 1 |  | 0 |  |  | Unclear |
|  |  |  | CIL | 6 | 8 | 5.2 | 12.5 | 1 |  | 0 |  |  | Unclear |
| **11** | [^18^F]PSMA-1007 | 312 | IIL | 4 | 5 | 3.4 | 7.8 | 1 | 826 | 0 | left | left | TP |
| **12** | [^68^Ga]Ga-PSMA-11 | 71 | IIL | 6 | 9 | 12.6 | 13.4 | 3 | 766 | 3 | left | none | TP |
| **13** | [^68^Ga]Ga-PSMA-I&T | 138 | CIR | NA | NA | NA | NA | NA | 813 | NA | both | right | FP |
| **14** | [^18^F]PSMA-1007 | 478 | CIL | 5 | 12 | 6.1 | 10.6 | 1 | 750 | 0 | left | left | Unclear |
| **15** | [^68^Ga]Ga-PSMA-I&T | 247 | EIL | 9 | 14 | 2.4 | 5.9 | 1 | 708 | 0 | left | left | FP |
|  |  |  | *Tr* | *5* | *7* | *NA* | *NA* | *NA* |  | *0* |  |  | *AD TP* |
| **16** | [^68^Ga]Ga-PSMA-I&T | 201 | Tr | 2 | 4 | 2.2 | 13.5 | 1 | 699 | 0 | none | none | FP |
| **17** | [^68^Ga]Ga-PSMA-11 | 174 | Tr | NA | NA | 3.9 | 14.2 | 1 | 783 | 0 | left | left | TP |
|  |  |  | CIL | 8 | 12 | 2.6 | 9.2 | 1 |  | 1 |  |  | FP |
| AD TP = additional true positive, CIL = common iliac left, CIR = common iliac right, EIL = external iliac left, EIR = external iliac right, FP = false positive, IIL = internal iliac left, IIR = internal iliac right, OE = other extrapelvic, RP = retroperitoneal, M1b = bone lesion, NA = not applicable, TN = true negative, TP = true positive, Tr = local recurrence | | | | | | | | | | | | | |
